# ‘Find Your Float’: A Public Health Approach to Addressing Drowning Inequities in People of African, Caribbean and Asian Heritage: A Cross-Sectional Study

**DOI:** 10.3390/ijerph23080990

**Published:** 2026-07-29

**Authors:** Heather Massey, Clare Eglin, Geoff Long, Danielle Obe, Ed Accura, Seren Jones, Alice Dearing, Ruth Williamson, Shelley Blane, Ross D. Pollock, Gareth Morrison, Michael J. Tipton

**Affiliations:** 1 Extreme Environments Laboratory, University of Portsmouth, Portsmouth PO1 2ER, UK; clare.eglin@port.ac.uk (C.E.); michael.tipton@port.ac.uk (M.J.T.); 2 QinetiQ, Portsmouth PO6 3RU, UK; 3 The Black Swimming Association (BSA), London WC2H 9JQ, UKseren.jones@thebsa.co.uk (S.J.);; 4 Hampshire Hospital NHS Foundation Trust, Basingstoke RG24 9NA, UK; ruth.williamson@hhft.nhs.uk; 5 School of Health and Rehabilitation Sciences, Bournemouth Campus, Health Sciences University, Bournemouth BH5 2DF, UK; shelley.blane@hsu.ac.uk; 6 Centre for Human & Applied Physiological Sciences, King’s College London, London SE1 1UL, UK; ross.pollock@kcl.ac.uk; 7 Royal National Lifeboat Institution, Poole BH15 1HZ, UK; 8 National Water Safety Forum, Birmingham B15 1RP, UK

**Keywords:** floating, drowning prevention, water safety, water immersion, aquatic activity

## Abstract

**Highlights:**

**Public health relevance—How does this work relate to a public health issue?**

- Globally, drowning is a preventable public health issue with high mortality and years of life lost.
- Where evidence is available, there appears to be a disproportionately high drowning risk among people from minority ethnic communities, highlighting inequities in exposure, access, and outcomes.

**Public health significance—Why is this work of significance to public health?**

- This work addresses floating as a key survival behaviour, and challenges long-held beliefs (e.g., ‘Heavy bones will make me sink’) that may limit engagement with aquatic activity and water safety practices.
- It also provides evidence to inform inclusive interventions targeting people from African, Caribbean and Asian communities, improving equity in prevention strategies.

**Public health implications—What are the key implications or messages for practitioners, policy makers and/or researchers in public health?**

- This work supports the concept of combining campaigns and community-based, co-produced interventions to improve in water floating competency.
- It highlights the need for accessible, community-based practice opportunities in addition to the risk communication and behavioural guidance that is already offered by current campaigns.

**Abstract:**

Drowning is a largely preventable global public health problem. Many unintended drownings are linked to the initial responses to cold-water immersion (cold shock), which includes a rapid involuntary cardio-respiratory response that increases the risk of aspirating water within the first two minutes of immersion. Floating rather than struggling to swim can help survival in this circumstance. In light of early research and subsequent changes in anthropometry, we are re-examining whether bone mineral density (BMD) in people from African, Caribbean, and Asian communities influences their float. This study examined predictors of float outcome. A total of 101 participants of African, Caribbean, Asian, or mixed heritage were recruited; 96 attempted a two-minute supine float following instruction and practice, of whom 89 were successful. Body fat percentage strongly correlated with buoyancy and floating outcome, while other predictors, including BMD, showed weak or no association. Passive floaters (no movement required) demonstrated greater buoyancy and less exertion than active floaters (purposeful movement required). These findings suggest that body fat percentage is the primary factor influencing float outcome, and that with appropriate instruction and practice, individuals of African, Caribbean and Asian heritage can “find their float”. The results indicate that floating is a learnable competence and highlight the need for integrated drowning prevention strategies combining existing campaigns with accessible, community-based practices and co-produced approaches to improve engagement and equity.

## 1. Introduction

The World Health Organization recognises drowning as a preventable global public health issue which affects all communities; an estimated 300,000 people die from drowning each year [1]. Between 2013 and 2023, an average of 20,181 people died annually from unintentional drowning in Europe, including 317 deaths in the UK [2,3]. Drowning disproportionately affects younger, healthier individuals; for this reason, it is suggested that Years of Life Lost (YLL) [4] should also be considered, as the measure captures the impact of premature death and may better reflect the impact on society. Globally, drowning has remained the third leading contributor to YLL, and disability adjusted life years from all unintentional injuries behind road traffic accidents and falls since records were published in 1990 [5,6]. Between 2013 and 2023, drowning accounted for an annual average of 18,511,673 YLL globally, with approximately 13,924 YLL recorded each year in the UK [2].

There are limited data on the ethnicity of people who have drowned. Where data are available, they indicate overrepresentation of people from minority ethnic communities [7]. For instance, in the United States, non-fatal drowning rates are approximately four times higher among children from African American and Hispanic communities compared with White children [8]. In New Zealand, people from Māori and Pacific islander communities [9] and Aboriginal people in Canada are overrepresented in the drowning statistics [10]. Furthermore, in Australia, people from Aboriginal and Torres Strait Islander communities were 1.7 times more likely to drown than non-Indigenous Australians [11]. Similarly, UK data suggest that the risk of drowning is around three and a half times higher in children from Black communities than in children from White communities [12].

In cold water, it is thought that many of the unintended drowning deaths are linked to the initial physiological responses to sudden immersion, known as the cold shock response [13,14]. The respiratory components of the cold shock response (CSR, including a 2–3 L inspiratory gasp and hyperventilation) increase the risk of water aspiration [14]. The lethal dose of fresh water for drowning is 44 mL.kg^−1^ and half that for sea water [15,16]; the initial gasp, if underwater, could be enough to start the drowning process. Pool-based studies indicate that on immersion in cold water the risk of drowning is increased if an individual tries to swim in the first minute of immersion when the CSR is at its peak [17]. Therefore, to reduce the risk of drowning, following sudden cold-water immersion, individuals should remain at rest, floating until they regain control of their breathing.

Immersion in cold water also provokes instinctive activity (swimming, waving, thrashing around) that increases the risk to the airway and cardiovascular system [18]. Fighting this instinct and staying still would therefore also be advantageous. In this case, floating in water out of a person’s depth necessitates floating on the back to keep the airway clear of the water; for some this will require little movement, for others it will require their head to be tilted backwards as well as sculling and kicking actions to maintain the airway at the surface of the water. Minimising movement during floating when clothed maximises the amount of air that remains trapped in clothing layers and increases buoyancy [19], thus reducing the effort needed to float. These studies are the basis of the water safety campaigns of ‘Float to Live’ [20] in the UK and similar subsequent initiatives in other countries (e.g., ‘float to survive’ in Australia). Research in realistic settings (calm and moving open fresh water and sea water) with inexperienced participants has supported and strengthened the advice to float on your back upon initial immersion [21]. The origins of the science and public health messaging around this are explained in greater detail by Tipton et al. [13].

Individuals who attribute their survival to public health campaigns promoting floating during cold-water immersion have subsequently provided unsolicited testimony regarding their experiences [22]. However, there are people who believe or report that they cannot float. This is believed within African and Caribbean communities and beyond, because their greater bone mineral density (BMD) (“heavier bones”) prevents floating [23]. Whilst higher BMD values in people of African and Caribbean heritage are well evidenced [24,25], it is not the case in Asian populations [26]. Consequently, the assumption that high BMD prevents floating and swimming is only valid if bone density is the sole or primary determinant of the outcome of these activities. The buoyancy provided by body fat could be a key determinant of the capability to float and swim. In the 1950s, it was reported that participants from Black communities have a lower mean body fat percentage (4.6%) than white participants (7.4%) [27]. The combination of low body fat and high bone mineral density may have made floating and swimming tasks more challenging [28]. However, since these papers were published 55 to 70 years ago, the mean body fat percentage of the UK population has increased to 24.4 ± 5.5% in males and 35.5 ± 6.7% in females [29] according to data collected up to 2010. By 2018, body fat percentage among individuals of African heritage in the United States had increased, reaching a mean of 25.5% (95% CI: 24.3–26.6%) in males and 37.1% (95% CI: 35.7–38.4%) in females [30]. This increase in body fat may result in an increase in buoyancy and enable individuals to float with greater ease than previously thought.

It is important to distinguish between the anthropometric variables that enable a body to float and the capability to adopt and maintain an effective floating position. Individuals with limited water competence, particularly when distressed, may be unable to position themselves appropriately despite having sufficient buoyancy to float. For many people, swimming and floating are both physically demanding and technically complex, requiring practice and competence. Opportunities to practice floating are often limited, even within swimming lessons. These challenges can be exacerbated by broader structural and cultural factors. In the UK and much of the Western world, aquatic and water-sport environments have historically been, and continue to be, shaped by unequal access, cultural norms, and socioeconomic barriers that affect participation and confidence in the water for people from minoritised ethnic communities [31,32,33,34]. Social and environmental factors such as few role models, including swimming athletes and swimming teachers/coaches, fewer opportunities to learn to swim, and reduced familiarity with aquatic settings can compound difficulties in accessing the practice needed to develop floating competency. Yet, with support (opportunity and access, use of water safety campaign materials, practice and instruction from a swimming teacher), participants who were initially not confident in their capability to float have been reported to be able to improve their float in a range of fresh and salt, moving and still water environments [21]. While floating practice is ideal and enables individuals to “find their float” (the technique that minimises the effort they need to stay afloat), opportunities to practice may not always be available or practical. Therefore, it is important to understand the factors that influence flotation and to provide clear guidance and instruction on how a person could float if they were unintentionally immersed in water.

This research aims to enhance representation of African, Caribbean and Asian communities in the water safety literature and inform education and awareness initiatives to reduce preventable drownings. We hypothesised: 1. The technique and effort needed to stay afloat when immersed in water can be characterised in people of African, Caribbean and Asian heritage. 2. Body fat levels rather than BMD predict floating outcome. 3. Buoyancy values will predict floating technique, with those who are less buoyant requiring more activity and greater effort to stay afloat than those who are more buoyant.

## 2. Materials and Methods

### 2.1. Design

This observational cross-sectional study started recruitment on 7 May 2024, and data were collected at Downside Fisher Youth Club in London and at King’s College London between 6 June 2024 and 12 December 2024.

### 2.2. Participants

Volunteers aged 18 to 60 years were recruited in response to advertising by the Black Swimming Association (BSA). Members of the general population who identified as being of African, Caribbean, Asian or mixed heritage provided their written informed consent to participate in the ethically approved study (Hampstead NHS Research Ethics Committee [23/SC/0414], UK). All testing was conducted in accordance with the principles of the Declaration of Helsinki.

Staff and members of the Black Swimming Association were embedded within the research team from its conception and study planning through to recruitment, supporting participants to float during data collection and reporting.

**Figure 1:**
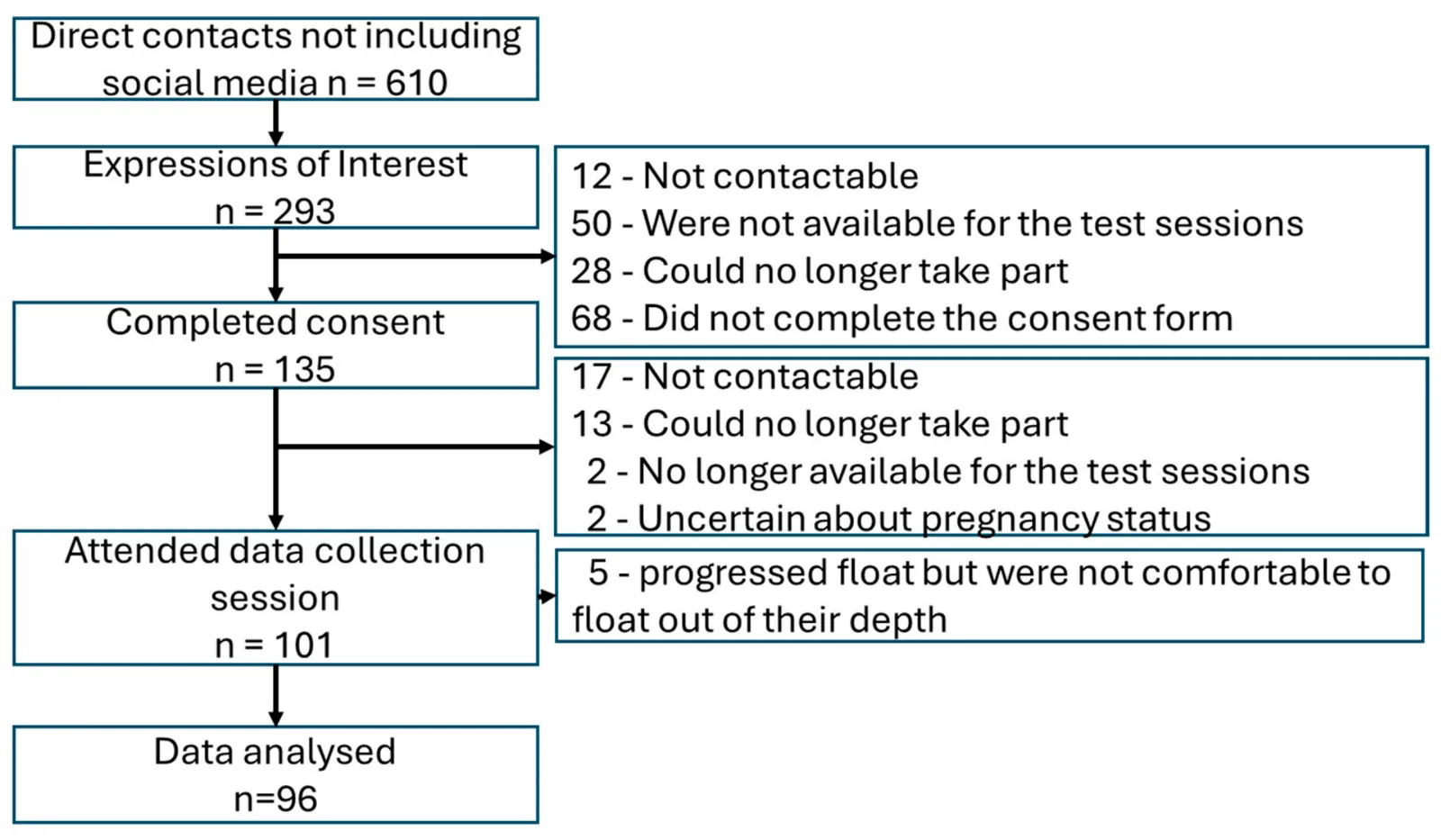
Participant flow through the study.

The participants’ flow through the study is shown in Figure 1. A total of 101 people took part in a float practice session. Five participants did not feel comfortable floating in water out of their depth and did not take part in further data collection. Participants were reimbursed for their travel expenses, but no gratuity for participation was provided.

### 2.3. Procedures

Participants completed a short survey about their current participation in water-based activities, whether they had ever attended swimming lessons, and to indicate their current swimming capability. Participant demographics (age, sex, self-reported ethnicity) were also recorded, and anthropometric measures were collected. Accessible body measurements of height, mass, and body girths were recorded. As well as conducting Dual-energy X-ray (DXA) scans for BMD (hip and lumbar spine) and body composition analysis (whole body), and forced vital capacity (FVC) as a measure of lung volume.

Participants practised their floating technique with an experienced instructor within and out of their depth for between 30 and 120 min until they were comfortable to undertake their recorded float in deep water. The practice and testing were conducted in a swimming pool filled with fresh chlorinated water, varying in depth from 1 to 2 m at a water temperature of 29.5 ± 0.6 °C.

#### 2.3.1. Float

Ninety-six participants completed a two-minute back float in a deep water pool. They wore their choice of swimwear (swimming costumes, shorts, or shorts and a T-shirt), with clothing prewetted and patted down to minimise buoyancy from trapped air; goggles or a dive mask were optional. Before floating, participants were fitted with a wrist-worn heart rate monitor (Forerunner 35, Garmin, Olathe, KS, USA) and reported their perceived exertion (RPE 0–10 scale [35]). They were instructed to “stay afloat on your back for two minutes using the minimum activity needed”. A lifeguard was ready to support the participant if required.

Each float began from the pool ladder: participants stepped down, adopted a squat, immersed to shoulder depth and gently pushed away from the ladder. The float was recorded from above, the side, and underwater using three synchronised IP68-rated static cameras (5 MP BarlusCam, Shenzhen Zhiyong Industry Co Ltd., Shenzhen, China) and one mobile waterproof camera (Olympus Tough TG-6 4K, Hachioji, Tokyo, Japan). Video was synchronised in OBS Studio (30.0.1) for analysis of float effort, posture, and technique (Supplemental Materials Table S1.1). If participants drifted toward walls or steps, they were repositioned for safety. Immediately afterwards, participants rated the effort required (RPE scale [35]) and described the confidence and difficulty of their float.

#### 2.3.2. Hydrostatic Weighing to Determine Buoyancy

Underwater weighing (hydrodensitometry) was used to estimate buoyancy in the same pool, on the same day as their float. The order of buoyancy and float data collection was dictated by equipment availability rather than assigned.

Participants lay in a supine position on a floating bed with their umbilicus at the midpoint of the bed. The bed was connected to a central base unit via a submersible load cell (Applied Measurements Limited, DBBSUB S-Beam Submersible Load Cell, DBBSUB-500N-006-000). Participants took a maximal inhalation, held onto the bed and submerged themselves at the minimum depth required for complete submersion for 20 s. The force required to submerge the participant and keep them submerged was measured continuously and transmitted wirelessly to a receiver attached to a laptop away from the poolside, which recorded load at 2 Hz. The depth of the bed was recorded at the participants’ head and feet to the nearest cm using a plastic ruler (mean depth at the head 19.7 cm, mean depth at the feet 26.8 cm).

Whilst lying supine on the floating bed, participants removed trapped air from clothing or swimming caps. To reduce anxiety and maximise the potential for quality measurements, participants were familiarised with the equipment and the procedure and gradually built up to the 20 s breath hold. Three 20 s breath holds were recorded to the nearest 0.01 N and averaged. Readings were made once the participant’s body was motionless and any air bubbles from the clothing and equipment had ceased. Water temperature was recorded using a waterproof thermometer (Zacro Aquarium thermometer, Shenzhen, China) which had been quality assured to be within 0.2 °C of a UK Standards Agency (UKSA) calibrated thermometer (T600 Digitron Precision Thermometer, Port Talbot, UK) at four water temperatures 28, 29, 30 and 31 °C prior to the start of the study.

Prior to, and at the end of every data collection session, the apparatus was calibrated with known weights (2 and 4 kg) and two sealed lightweight metal bottles (each containing approximately 1 L of air and providing 9.47 N of buoyancy). Additionally, the bed was weighed unloaded before and after each person had been measured.

#### 2.3.3. Body Composition

Anthropometric measurements of height (Seca 213, Seca, Hamburg, Germany), mass (M-420 portable scale, Marsden, Rotherham, UK), circumferences of the chest, waist, hip and thigh (W606PM, Lufkin, UK) were recorded in a private room following the procedures of Norton & Eston [36]. Measurements were recorded in duplicate, and if measures differed by greater than 5%, a third measurement was recorded. Six study team members completed the measurements and inter- and intra-rater reliability was assessed (Supplemental Material Table S1.2a–h).

Percentage body fat was calculated by impedance (Body stat 1500, Body stat Limited, UK), by whole body composition scan using a DXA-scan (Hologic Horizon A Densitometer, Hologic, Bedford, MA, USA) and using a more accessible means of calculating relative fatness equation (RFE) from height and waist circumference [37].

Each participant’s BMD was measured at the left hip and the lumbar spine (L1–L4) using the DXA-scanner. Prior to the scans, all participants were screened to ensure the scan would not be contraindicated (e.g., due to suspected pregnancy) and to prevent an unnecessary dose of ionising radiation (e.g., in the case of hip replacement surgery). Participants’ position was optimised in accordance with the manufacturer’s guidance for each scan. Scans were analysed in accordance with standard practice from the manufacturers and reported by the radiographer (RW). Where Osteopenia (T-score −1) or Osteoporosis (T-score −2.5) was suspected (*n* = 7), participants were contacted, and where they provided consent, their General Practitioner was contacted.

#### 2.3.4. Spirometry

Forced vital capacity (FVC) was measured using flow volume loops (Spirobank, MIR, Roma, Italy) in accordance with the guidance of the American Thoracic Society and European Respiratory Society [37]. Participants had a minimum of 1 min to rest between each manoeuvre and were coached to optimise technique using appropriate demonstrations and phrases [38]. Participants completed the manoeuvres in a standing position wearing a nose clip or tightly pinching their nose if the nose clip would not stay on their nose. The three manoeuvres meeting the guidance were recorded, and the mean of the three was reported. Where there was an incidental finding, participants with diagnosed airflow limitation (*n* = 3) were advised to follow their prescribed preventer medication regimen and consult their doctor. Participants with no diagnosed respiratory disease (*n* = 2) were informed of the incidental findings and advised to contact their doctor; with consent, relevant findings were also communicated to their General Practitioner.

#### 2.3.5. Surveys

Before starting any floats or practice and after their recorded float, participants were asked how difficult they found floating (1 = very difficult, 2 = difficult, 3 = neutral, 4 = easy, 5 = very easy) and how much confidence they have in their capability to float (1 = very unconfident, 2 = unconfident, 3 = neutral, 4 = confident, 5 = very confident). After the float, they were also asked about their perception of floating compared to the start (1 = a lot more difficult, 2 = more difficult, 3 = about the same as they thought, 4 = less difficult, 5 = a lot less difficult). After the float, participants were asked what instructions and practices helped them to float and if their experience would increase the likelihood of them participating in water-based activities in the future.

#### 2.3.6. Interviews of Swim Coaches

Swim coaches were asked about the techniques they used to support people to learn to float. They were asked what their aim with each person was, what steps they followed when instructing people, how long they supported participants during the trial, and what the barriers and facilitators to floating were.

### 2.4. Data Analyses

Heart rate measurements were logged continuously and recorded at 30 s intervals. The description of the participant’s floating technique was made using a checklist (available in the Supplementary Materials). The final 30 s of each participant’s float was assessed for airway and neck position, trunk angle, level of tension, arm and leg movements, coordination and technique, frequency and intensity of movement, work–rest pattern and float outcome using this checklist. If it was not possible to assess the final 30 s of the float (due to them floating out of camera view), the final 30 s the participant was visible in the footage was used (*n* = 2). Each video was assessed by two researchers (HM and CE) simultaneously to come to a consensus regarding float topography (both HM and CE have previous experience in assessing float technique [21] and have good inter-rater reliability with other international researchers [unpublished data]). In addition to the checklist, the passive or active nature of the float was determined in accordance with Eglin et al. [21] based on arm and leg movements in the final 30 s of the float. ‘Active’ floaters required purposeful movement to maintain their airway above water; ‘passive’ floaters used limb movement for stabilisation only, if at all. In addition, the shape adopted in the water was also described. Representative images of the different postures adopted were recreated using ChatGTP 5.5. based on images recorded during data collection. 

Still profile images of the participants’ body position in the water were also recorded for later analysis of the participants’ trunk angle (Quintic Biomechanics v33, Quintic, Birmingham, UK) when floating. The lip of the pool was used as the horizontal reference and a straight line from the centre of the deltoid to the iliac crest of the hip as the trunk position.

### 2.5. Statistical Analyses

Statistical analyses were performed using Prism GraphPad 10.4 (GraphPad, Boston MA, USA) and SPSS v 29 (IBM) and RStudio v 4.6.1. Parametric analyses were used for normally distributed interval and ratio data, including anthropometric and lung volume measures. Non-parametric analyses were used for non-normally distributed, ordinal, or nominal data, including heart rate, confidence, perceived difficulty, rating of perceived exertion (RPE), and self-reported swimming ability. Fleiss’ kappa (κ) was used to analyse inter-rater reliability for the collected anthropometric data and weighted Cohen’s κ for float description scales. Relationships between metrics were investigated using Pearson’s correlation. Within-participant comparisons were conducted using paired-samples *t*-tests or Wilcoxon signed-rank tests, while between-group comparisons were conducted using independent-samples *t*-tests or Mann–Whitney *U* tests, as appropriate. Fisher’s Exact test was used to examine the relationship between those attending swimming lessons and those who did and did not float.

Linear (for heart rate data) or ordinal mixed effects model (for RPE, confidence and difficulty ratings) compared characteristics of active and passive (float) groups at the start and end of the floats. Limits of agreement [39] were also used to assess the level of agreement between body fat percentage values obtained from BIA, DXA, and the RFE [37]. The association between float outcome and anthropometric/physiological measures was examined using ordinal logistic regression. Two models were produced, one using specialised body composition measures (such as DXA scan variables) and the other using pragmatic accessible measures (e.g., RFE). Variables exhibiting substantial multicollinearity were assessed using correlation coefficients and variance inflation factors (VIFs). Where multiple variables measured the same construct (e.g., body fat assessed by DXA, RFE calculations, and bioimpedance), the most accurate and physiologically relevant measures were retained to minimise redundancy and model instability.

Mediation was examined using structural equation modelling implemented in the *lavaan* package in R. Measures output from the logistic regression were used as covariates, buoyancy as the mediator, and float outcome as the ordinal outcome. Two models were created, one using specialised body composition measures, the other for pragmatic accessible measures.

Values are presented as mean (standard deviation) unless stated otherwise; the threshold for significance was taken as *p* ≤ 0.05 and thresholds for the strength of correlation were considered as 0.2 weak, 0.5 moderate, 0.7 strong correlations [40]. Effect sizes were also calculated: Hedges’ g using the average variance standardiser rather than the pooled standard deviation following *t*-tests, *r* = Z/√N following Wilcoxon and Mann–Whitney *U* tests, and odds ratio (OR) following ordinal mixed effects models and ordinal logistic regression. Thresholds for Hedges’ *g* were <0.2 trivial, 0.20–0.49 small, 0.5–0.79 moderate and 0.8 large; the thresholds for *r* were 0.1 small, 0.3 medium, and 0.5 large.

## 3. Results

### 3.1. Participants

A total of 96 participants (43 male, 53 female) volunteered to float out of their depth in a heated indoor pool. Ethnicity, personal characteristics, and distributions of physical characteristics are provided in Table 1 and Table 2 and Supplementary Figures S1.1 and S1.2. Of the cohort, 73 participants (76%) had received swimming lessons, while 23 (24%) had not. 75% reported no or minimal recent aquatic experience (Table 3). Participants reported a median swim capability of 6 (IQR 4–8) on a 1–10 scale (1 = cannot swim; 10 = excellent swimmer), with 33 individuals (34%) rating themselves between 1 and 4.

### 3.2. Float Descriptions

Ninety-six participants felt confident attempting a two-minute deepwater float after one-to-one instruction. The mean float time was 112 ± 27 s. Eighty-seven participants completed the full 120 s, two stopped early for reasons unrelated to floating competence, and seven self-rescued or were assisted after floating for 0–75 s.

During the final 30 s of each participant’s float, the postures adopted and actions performed during the float were recorded and are summarised in Table 4 and Table 5 and Supplemental Materials Tables S2.1 and S2.2. Several participants adopted more than one activity level or changed their posture during the final 30s of the float; all postures and activities have been recorded; therefore, the activities or postures may add up to greater than the total *n* of 96. The most common float position for participants who required little or no effort to stay afloat had their head back, with the airway never submersed or only occasionally, with the majority either adopting a relaxed or neutral body tension and no or limited arm and leg movements. For participants who needed to be more active in their float (float with some effort or with lots of effort), their neck was extended so that their ears were immersed in water, bodies tense or rigid, with frequent, moderate- to high-intensity arm movements, and some leg movement. Of the 60 recorded arm actions (from 46 participants), all were synchronised, 12 adopted a sculling action, 18 used circles, 18 pushed down from the water surface, and 12 used a sweep or flapping action. Participants tended to use kicking less (23 participants); 19 adopted a cycling kick, 6 a flutter/flick kick and 2 a synchronous breaststroke kick; most participants did not move their legs (*n* = 73).

Where float angle could be recorded (*n* = 86, mean float angle 44.8° ± 20.1°), 28 (29.2%) adopted a trunk angle between 0 and 29.9° (horizontal to the water surface), 41 (42.7%) an angle between 30 and 59.9° (diagonal to the water surface) and 17 (17.7%) between 60 and 89.9° (vertical). It was not possible to accurately record float angle in 10 (10.4%) participants.

**Figure 2:**
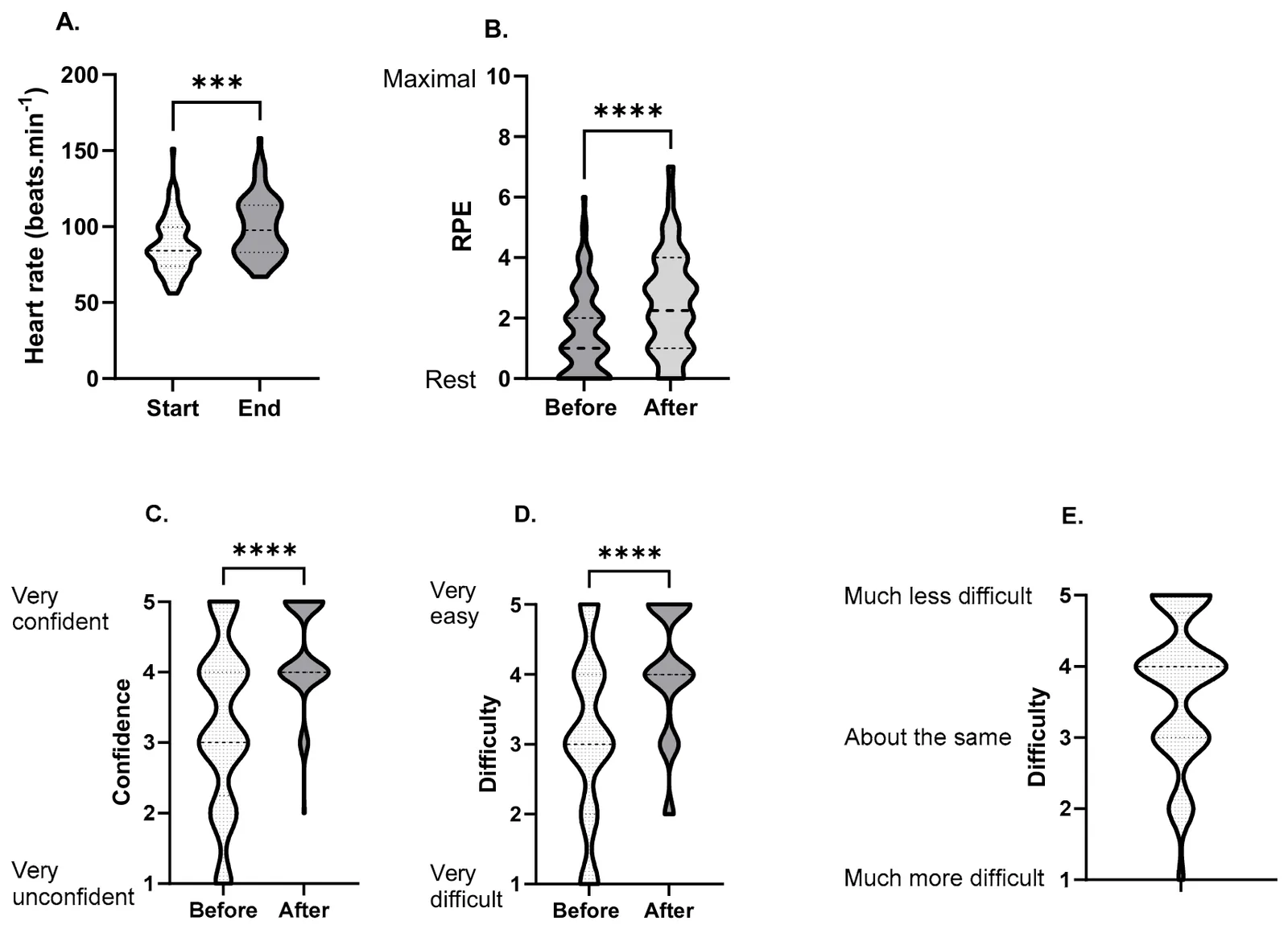
Responses before, during, and after a two min float in calm pool water. (**A**) Heart rate at baseline before the start of the float and end of the float, (**B**) rating of perceived exertion, (**C**) confidence, (**D**) difficulty before and immediately after the float, and (**E**) the perception of difficulty in response to the question: How did floating compare to how difficult you thought it would be? The width of the violin plots indicates the frequency of votes. Bars indicate significant difference, *** *p* < 0.001, **** *p* < 0.0001.

Heart rate at the end of the two min float was significantly higher compared to the start (start: median 87 [range 56–151] beats.min^−1^; end: median 95 [range 67–158] beats.min^−1^, *W* = 2663, *p* < 0.001, Figure 2A). Similarly, RPE was significantly greater after the float (median 3 = moderate [range 0–7]) compared to before floating (median 1 = very, very easy [range 0–6], *W* = 2442, *p* < 0.0001, Figure 2B). Participants’ confidence in their capability to float increased following their float practice and two min float (from 3 = neutral [1,2,3,4,5] to 4 = confident [2,3,4,5], *W* = 2034, *p* < 0.0001, Figure 2C) and they perceived the float to be easier (before 3 = neutral difficulty [1,2,3,4,5], after 4 = easy [2,3,4,5], *W* = 2157, *p* < 0.0001, Figure 2D). As a result, the float was perceived as less difficult than expected (median 4, Figure 2E).

**Figure 3:**
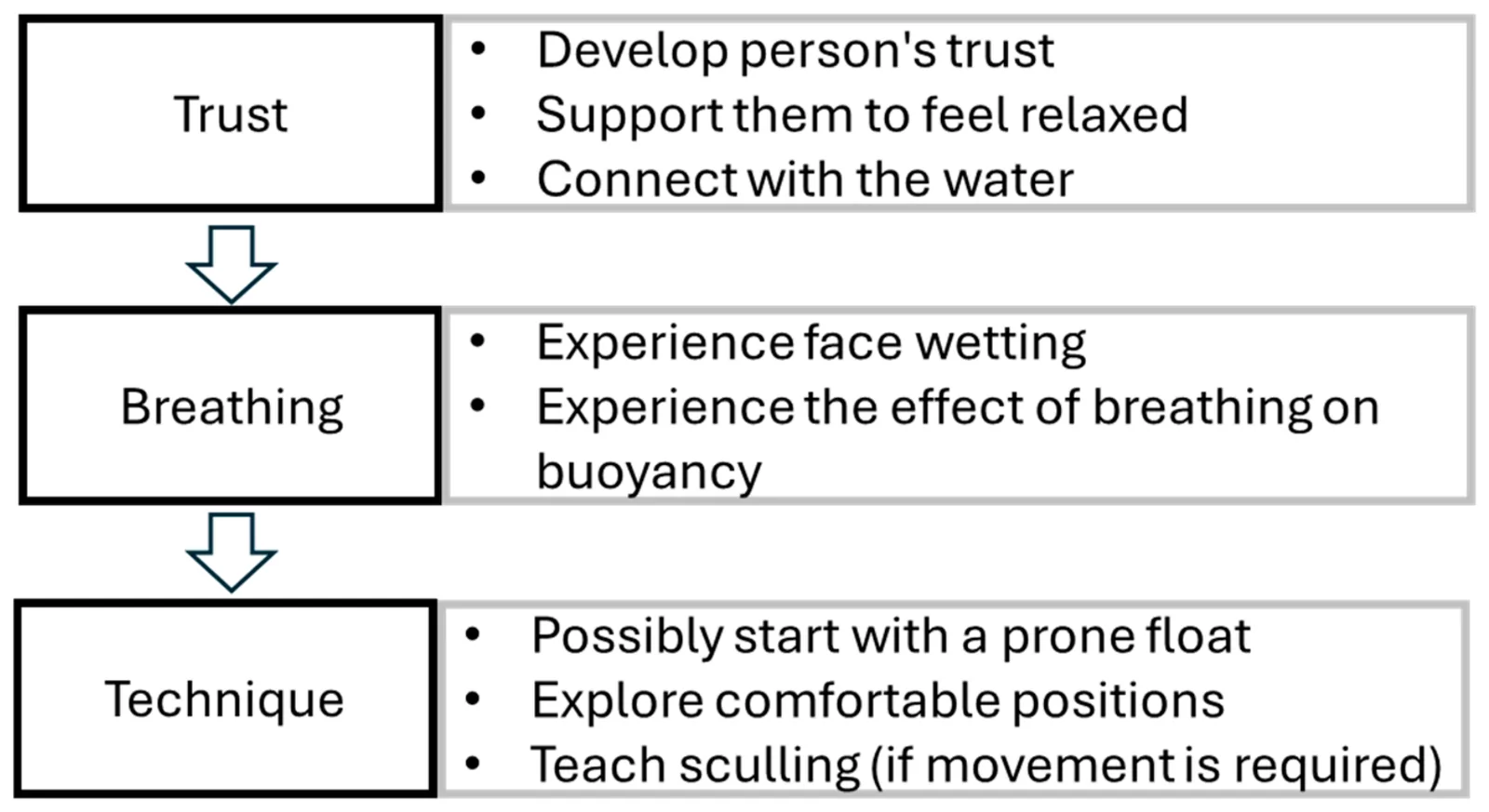
Instructors’ process of supporting participants to float; a narrative account of this process can be found in the Supplemental Materials.

The participants found these instructions most helpful for floating: to relax (*n* = 51); put their head back (*n* = 50); and maintain shallow, consistent breathing/not breathing in or out too deeply or quickly (*n* = 49). Instructions on arm activity were found helpful by 34 people, while 23 found the guidance on leg movement helpful. Fourteen participants found it useful to try different floating positions/shapes, including floating in a prone position first; in addition, 11 participants suggested that practice was helpful, or they recognised that they were overthinking the activity, or they needed to remain calm. Seven participants explicitly mentioned that it was helpful to move, whether it was their location in the water or moving their arms or legs. The instructors also provided insights into the processes they used to support participants to float. An overview of their processes is provided in Figure 3, and a narrative account is provided in the Supplementary Materials.

### 3.3. Characteristics of Individuals Who Floated Versus Those Who Did Not

The characteristics of the seven men who did not complete the two-minute float were compared with those who did (Table 6). Although the non-floating group had significantly lower height, body mass, BMI, and lean mass, all values, except one BMI, fell within the range observed among the men who successfully floated.

### 3.4. Factors Associated with Floating and Actions to Float

Correlations were examined between float outcome, buoyancy, and floating descriptions (Table 7 and Table 8 and Supplemental Figures S1.3 and S1.10). Float outcome showed strong positive correlations with buoyancy. Both measures were strongly negatively correlated with arm movement frequency, intensity, and arm work–rest ratios, and moderately negatively correlated with leg activity. Trunk angle showed weak but significant correlations with both float outcome and buoyancy.

Associations between float outcome or buoyancy and body composition measures were examined. BMD, lean mass, and lung volume showed no or weak correlations with float outcome and buoyancy. In contrast, body fat percentage showed moderate-to-strong positive correlations with both float outcome and buoyancy. In addition, change in heart rate (from float start to end) was weakly to moderately correlated with buoyancy (*r* = −0.364, *p* < 0.001) and float outcome (*r* = −0.556, *p* < 0.001).

Correlations between body tension, limb activity, and body composition measures showed that greater relaxation was associated with lower arm movement frequency and intensity and with higher body fat percentage, especially in the gynoid/android regions, but showed no or weak correlations with lean mass or BMD. Overall, lower RPE at the end of the float was correlated with greater buoyancy, increased relaxation, reduced arm activity, and less effort required to stay afloat (lower float outcome values).

### 3.5. Regression Analysis

**Figure 4:**
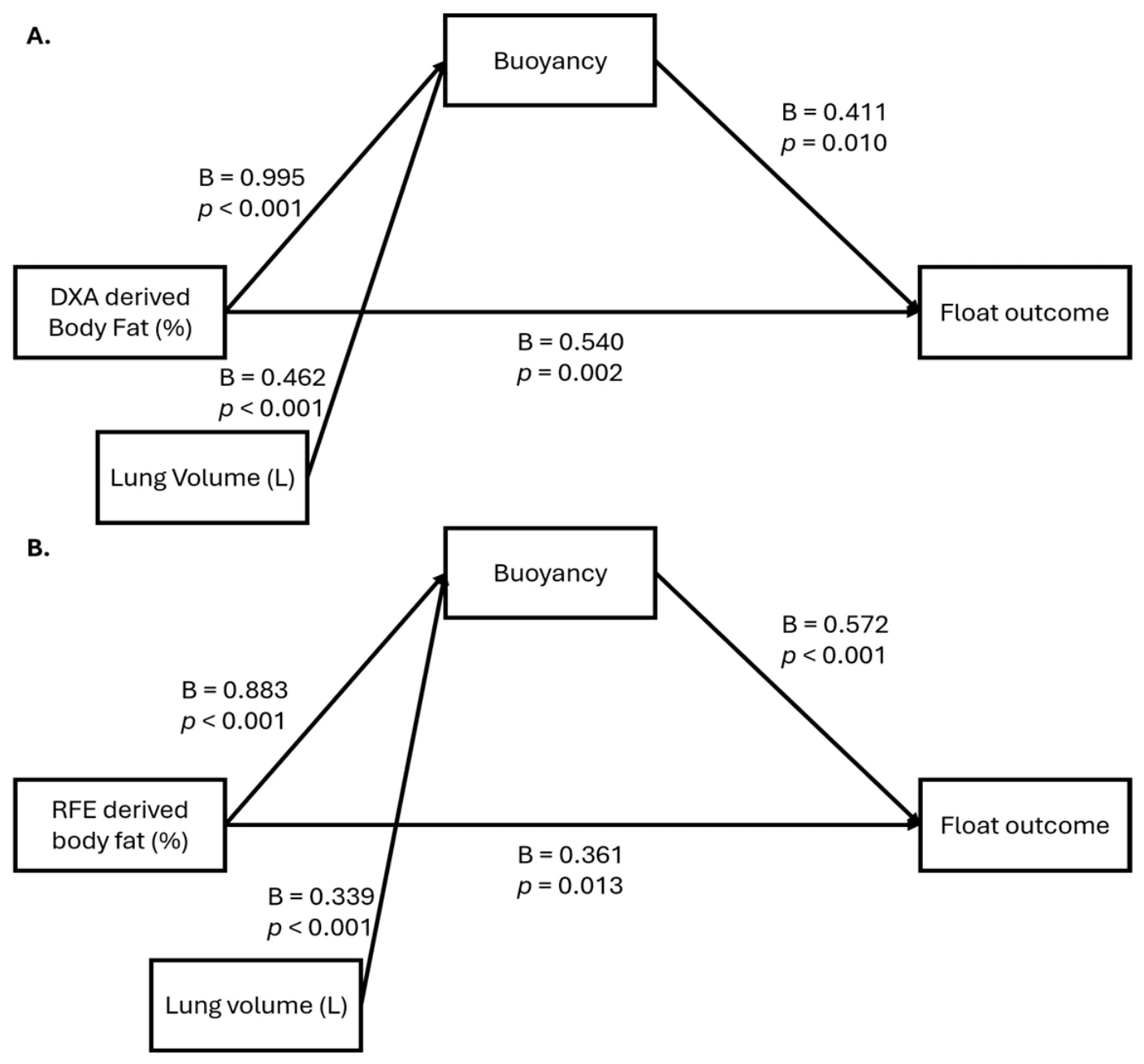
Mediation diagrams showing that buoyancy partially mediated the relationship between float outcome and (**A**) specialised measures; (**B**) RFE, more accessible measures [36].

Given the large number of correlations performed, the risk of Type I error was elevated. To strengthen the findings and triangulate results, additional analyses of regression and mediation (Figure 4) were used to further assess the relationship between float outcome and body composition.

#### 3.5.1. Regression Model Using Specialised Measures of Body Composition

Variables of BF_(DXA)_ (%), lean mass (kg), BMD and FVC were retained for the regression. The model significantly improved prediction compared with an intercept-only model, χ^2^(4) = 63.26, *p* < 0.001, explaining 51.8% of the variation in float outcome (Nagelkerke R^2^ = 0.518). Greater fat mass (*β* = 0.215, OR = 1.24, 95% CI [0.143, 0.288], *p* < 0.001) and greater FVC (*β* = 0.766, OR = 2.15, 95% CI [0.256, 1.275], *p* = 0.003) were associated with increased odds of belonging to a float outcome category characterised as requiring less effort to float. Higher lean mass was associated with lower odds of belonging to a float outcome category characterised by lower effort requirements (*β* = −0.097, OR = 0.91, 95% CI [−0.160, −0.034], *p* = 0.002). BMD was not a significant predictor (*p* = 0.242). The proportional odds assumption was violated, χ^2^(12) = 23.48, *p* = 0.024, suggesting that predictor effects may vary across floating outcome thresholds; therefore, the estimated odds ratios should be interpreted with caution.

#### 3.5.2. Regression Model Using More Accessible Measures of Anthropometry

The RFE and FVC variables were retained for the regression. The model provided a significantly better fit than the intercept-only model, χ^2^(2) = 50.34, *p* < 0.001. The proportional odds assumption was met, χ^2^(6) = 2.19, *p* = 0.901 and demonstrated good fit to the data (Pearson χ^2^(378) = 298.22, *p* = 0.999; Deviance χ^2^(378) = 207.34, *p* = 1.000). Both predictors were significant. Higher relative fat mass (*β* = 0.233, SE = 0.038, OR = 1.26, 95% CI [0.159, 0.308], *p* < 0.001) and greater FVC (*β* = 0.747, SE = 0.221, OR = 2.11, 95% CI [0.314, 1.179], *p* < 0.001) were associated with greater odds of belonging to a float outcome category characterised by lower effort requirements. Relative fat mass exhibited the strongest association with floating outcome (FVC, Wald = 11.44; RFE, Wald, 37.63). The model explained approximately 43.8% of the variation in floating outcome (Nagelkerke R^2^ = 0.438).

### 3.6. Mediation Analysis Using Measures of Body Composition

#### 3.6.1. Mediation Including Specialised Measures of Body Composition

BF_(DXA)_ (%) and FVC were significant predictors of buoyancy (*β* = 0.955, *p* < 0.001 and *β* = 0.462, *p* < 0.001, respectively), whereas lean mass was not (*β* = −0.120, *p* = 0.177). Greater buoyancy was associated with improved floating outcome (*β* = 0.411, *p* = 0.010). Significant indirect effects were observed for both body fat percentage (*β* = 0.393, *p* = 0.009) and FVC (*β* = 0.190, *p* = 0.021), indicating that buoyancy mediated their relationships with floating outcome. Body fat percentage also retained a significant direct effect on floating outcome (*β* = 0.540, *p* = 0.002), suggesting partial mediation. The model explained 67.5% of the variance in buoyancy and 71.1% of the variance in floating outcome.

#### 3.6.2. Mediation Including More Accessible Measures of Body Composition

FVC and RFE significantly increased buoyancy, which in turn reduced the effort required to float (float outcome). RFE exhibited a particularly strong association with buoyancy (*β* = 0.883, *p* < 0.001), whereas FVC showed a more moderate effect (*β* = 0.399, *p* < 0.001). Buoyancy was a significant predictor of float outcome (*β* = 0.572, *p* < 0.001). The direct effect of FVC on float outcome was not significant after accounting for buoyancy (*β* = 0.149, *p* = 0.164), indicating that the influence of FVC on float outcome was fully mediated by buoyancy. In contrast, RFE retained a significant direct effect on float outcome (*β* = 0.361, *p* = 0.013), suggesting partial mediation. The model explained 52.4% of the variance in buoyancy and 66.4% of the variance in float outcome.

### 3.7. Passive v Active Floating

Participants were classified as either active (purposeful activity performed during the float) or passive floaters (no activity required to stay afloat) [21]; mean data are shown for the two groups in Table 9. All but two active floaters floated for a two-minute float; the two that did not stopped of their own accord, one due to the perceived proximity of their head to the wall, and the other reported hearing a loud noise and regained a treading water position.

Comparing the passive to active floaters, the passive floaters were shorter, had significantly less lean mass (both BMD and muscle mass), more body fat and smaller FVC. These differences in body composition led to greater buoyancy values in the passive floaters, and lower HR and RPE scores at the end of the float. Both groups floated with similar trunk angles (Table 9).

**Figure 5:**
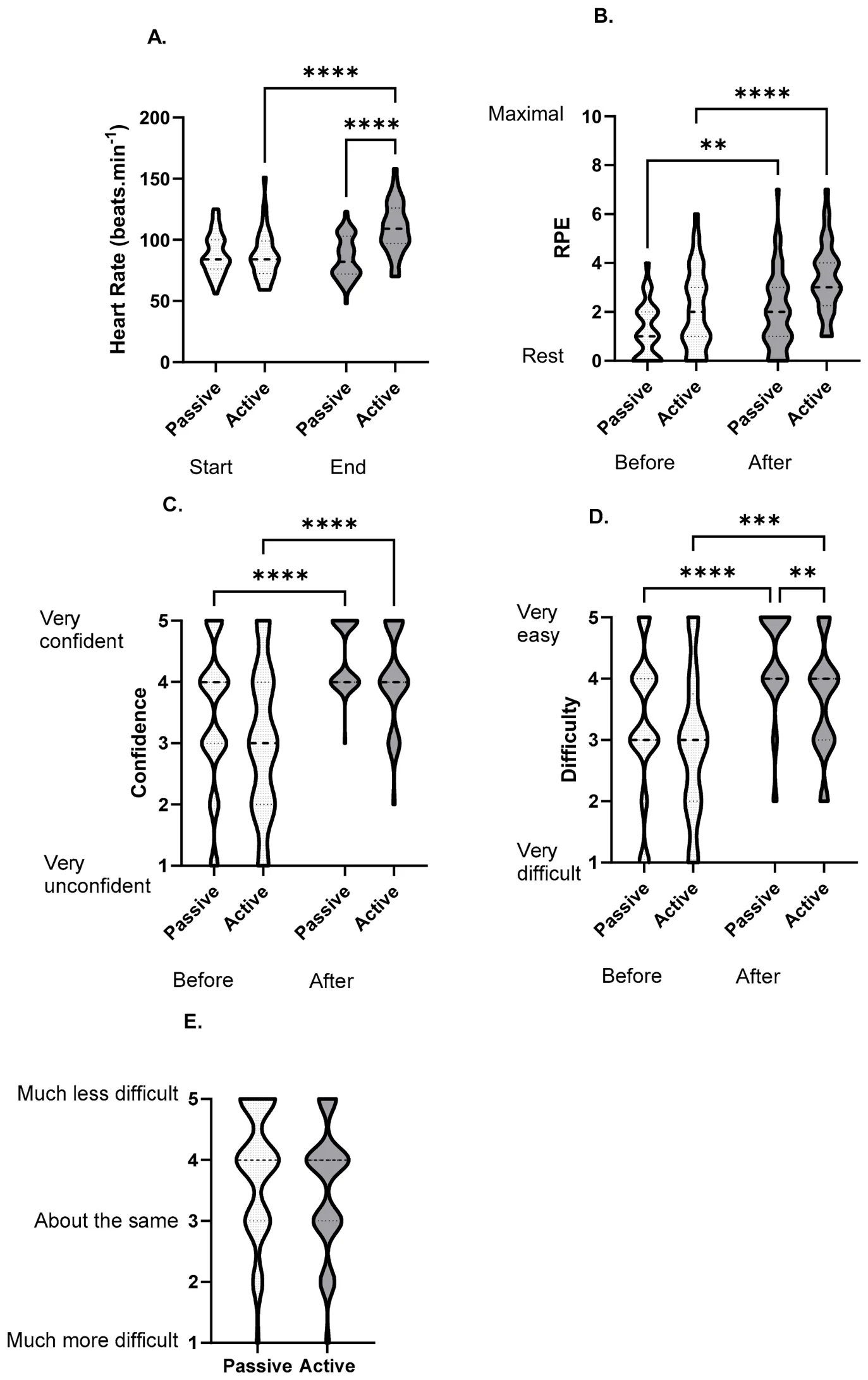
Responses to floating in participants who floated actively or passively. (**A**) Heart rate at baseline before the start of the float and end of the float, (**B**) rate of perceived exertion, (**C**) confidence, (**D**) difficulty before and immediately after the float, and (**E**) the perception of difficulty in response to the question: How did floating compare to how difficult you thought it would be? The width of the violin plots indicates the frequency of votes. Bars indicate significant difference, ** *p* < 0.01, *** *p* < 0.001, **** *p* < 0.0001.

When comparing HR between the start and end of the float and between active and passive floaters, there was a significant time × group interaction (*β* = −23.71, *SE* = 3.46, *t*(94) = −6.86, *p* < 0.001, *r* = 0.58), indicating that the magnitude of change in heart rate differed between groups. No baseline differences were observed between groups (*β* = 1.07, *SE* = 3.65, *t*(144.24) = 0.29, *p* = 0.769, *r* = 0.02). Active floaters demonstrated a significant increase in heart rate from start to end (*β* = 26.54, *SE* = 2.71, *t*(94) = 9.80, *p* < 0.001, *r* = 0.71). In comparison, there was no difference in HR in passive floaters from the start and end of the float (*β* = 2.83, *SE* = 2.15, *t*(94) = −1.32, *p* = 0.190, *r* = 0.13, Figure 5A).

There was no interaction effects for RPE between the passive and active float groups over time (*β* = −0.22, SE = 0.55, *z* = −0.40, *p* = 0.693, OR = 0.80) and no main effects of group (*β* = −0.29, SE = 0.45, *z* = −0.64, *p* = 0.522, OR = 0.75) but there were separate main effects of time with RPE increasing in both groups from the start of the float to the end (*β* = −1.24, SE = 0.36, *z* = −3.46, *p* = 0.001, OR = 3.47, Figure 5B).

There was no interaction effect of confidence (*β* = −1.01, SE = 0.58, *z* = −1.74, *p* = 0.083, OR = 0.36), or main effect of between groups’ confidence levels (*β* = −0.68, SE = 0.48, *z* = −1.41, *p* = 0.159, OR = 0.51), there was a significant main effect of time (*β* = 2.49, SE = 0.45, *z* = 5.57, *p* < 0.001, OR = 12.10). Both the passive and active groups increased in confidence by the end of the two min float (Figure 5C). Similarly, there was no interaction effect for float difficulty (*β* = −1.01, SE = 0.58, *z* = −1.74, *p* = 0.083; OR = 0.36), and no main effects of group (*β* = −0.68, SE = 0.48, *z* = −1.41, *p* = 0.159, OR = 0.51), but there was a main effect of time (*β* = 2.49, SE = 0.45, *z* = 5.57, *p* < 0.001, OR = 12.10, Figure 5D). The median response for both groups when reporting the perception of ease/difficulty of floating was ‘about the same’ level of difficulty or ‘less difficult’ than they thought (Figure 5E).

### 3.8. Agreement Between Body Fat Measurements

Agreement between body fat measurements derived from DXA was compared to RFE calculations and BIA measurements. Both methods slightly overestimate body fat percentage compared to DXA (RFE Bias 2.1%, BIA 3.2%). The 95% limits of agreement between DXA and RFE suggest that individual differences between the two methods could range from approximately −5.4% to 9.6%. The wider limits of agreement between BIA and DXA (–7.3% to 13.80%) reflect greater variability and less agreement than RFE and DXA (Supplementary Material Figure S1.11).

## 4. Discussion

This study has significant implications for public health and drowning prevention, particularly in addressing disparities in aquatic participation and water safety outcomes among African, Caribbean, and Asian communities. The findings contribute important evidence to the ongoing discussion regarding floating capability in populations of African and Caribbean heritage. It has previously been suggested that elevated bone mineral density (BMD) may negatively influence floating competence. However, it is important to recognise that participants in the present study differ anthropometrically from those examined in earlier studies; therefore, differences in findings may reflect differences in the populations studied. While high BMD values were observed in this cohort, they were consistent with established normative data [41,42,43,44]. Furthermore, BMD was not associated with float outcome, whereas body fat and lung volume explained 43.8–71.1% of the variance. Therefore, our hypotheses are accepted. Instead, floating outcome was primarily determined by factors including body fat and lung volume, which are mediated by buoyancy; other factors, such as technique and the ability to relax in water, may improve the model further.

Our participants included both men and women spanning a wide age range (19 to 60 years), with a mean Body Mass Index (27.7 kg.m^−2^) comparable to that of the general UK population [45] and of participants from previous floating research [20]. This is noteworthy given the substantial changes in population body composition that have occurred over the past 70 years. Although nationally representative BMI data are limited for the mid-twentieth century, obesity was relatively uncommon before the 1980s. Since then, obesity prevalence in England has increased markedly, rising from approximately 6% of men and 9% of women in 1980 to 27% and 29%, respectively, in 2019 [46]. These trends reflect a broader population-wide shift towards higher BMI and adiposity over time. As adiposity contributes to buoyancy, changes in population body composition over time should be considered when interpreting and comparing floating outcomes across studies conducted in different eras.

The observation that the majority of participants had limited recent exposure to water and low initial confidence suggests the sample of participants had similar experience levels to the participants of African, Caribbean and Asian heritage surveyed by Sport England [47], for whom participation in water-based activities was lower than in other communities. This further underscores the role of experiential inequity in shaping aquatic competence and confidence. Importantly, despite our participants’ limited recent water exposure and low initial confidence, most were able to achieve a two-minute float following instruction and practice, reinforcing the effectiveness of competence-based interventions.

These findings reframe floating as an acquired competence that can be learned by everyone, given the time to learn and practice. It also highlights the importance of promoting it as an activity that can be learned within water safety campaigns and through community-focused messaging. Beliefs such as the “heavy bones” narrative may function as culturally embedded barriers, discouraging engagement with aquatic environments and reducing opportunities for acquisition of potentially lifesaving competencies. In line with Wilcox-Pigeon et al. [7], these findings highlight the need to situate drowning risk within a broader public health framework that recognises the role of social determinants, including access to facilities, historical exclusion, cultural beliefs, and opportunities for learning. Addressing such determinants is essential to improving outcomes in populations that are disproportionately at risk.

Participants with lower buoyancy demonstrated greater physiological and perceived effort while attempting to remain afloat. Reduced buoyancy was associated with larger increases in heart rate during floating attempts and greater effort to maintain a stable position at the water surface. This suggests that while floating is achievable across a wide range of body compositions, individuals with lower buoyancy may experience the task as more physically demanding and may require additional practice to develop efficient techniques that minimise energy expenditure. From a drowning prevention perspective, this is important because elevated effort and physiological strain have the potential to increase fatigue and anxiety during an unexpected immersion. Risk communication should therefore emphasise that successful floating does not necessarily mean remaining completely motionless and that different individuals may need to adopt varying levels of movement and different body positions to remain afloat.

The principles underpinning the established “Float to Live” campaign [13] have been widely deployed as a population-level drowning prevention strategy in the UK and adapted in other countries (Australia and New Zealand). Nevertheless, while public safety campaigns are necessary, for communities with limited prior exposure to water, additional support may be necessary to provide the experience and opportunities to practice that are needed to improve floating competence. A further key finding with direct relevance to risk communication is the observed discrepancy between perceived and actual competence. Although confidence increased following participation in the floating practice, this did not always correspond to improved capability. Notably, the seven male participants who required more practice to float still reported feeling neutral or confident in their capability to float following their attempt. This reinforces the fact that increased confidence without corresponding competence may elevate risk exposure [48]. Public health messaging should therefore focus on building confidence that floating is achievable when appropriate instructions are followed, while also emphasising that floating competence develops through learning, practice, and experience.

These findings support the implementation of interventions that integrate public health and safety communication with community-based, experiential learning. At the population level, existing campaigns such as “Float to Live” should be maintained and extended, with adaptations to explicitly address cultural beliefs and to emphasise that floating is achievable across diverse populations. Messaging should recognise that individuals may require varying levels of movement and technique to remain afloat, depending upon their body size and composition. This should also reduce the perception of a single “correct” floating posture and promote awareness of the range of body positions, activity levels and techniques that could be used (Table 4), increasing the likelihood of individuals successfully ‘finding their float’.

Wilcox-Pigeon et al. [7] suggest that campaigns to support drowning prevention strategies must be complemented by accessible, in-person interventions. The present study demonstrates that co-production with community representatives is essential to the design and delivery of both communication strategies and practical interventions. Engaging communities in this way enhances cultural relevance, builds trust, and improves the likelihood of sustained engagement. This will involve collaboration with representative community leaders and instructors, and the development of locally tailored messaging and delivery models.

Finally, while ‘finding your float’ represents one water competence critical to survival when immersed in water, the study reinforces the need for comprehensive water safety education that extends beyond acquisition of discrete competencies. Experimental conditions in this study did not replicate real-world immersion scenarios, particularly open water exposure, where conditions such as wind and waves need to be, and can be, experienced [21,49]. Risk communication must therefore incorporate hazard awareness, environmental knowledge, and behavioural strategies to mitigate risk in realistic conditions to help prevent drownings [50].

### Strengths and Limitations

The use of high-precision measurement techniques was a strength of this work. However, given the strong agreement between RFE and DXA and the weak association between BMD and float outcome or buoyancy, future studies investigating flotation may not require the use of high-cost, high-precision methods such as DXA. More accessible, affordable, and time-efficient measures of body composition, such as RFE [37], may be sufficient for characterising body fat in research focused on floating competency and water safety.

**Figure 6:**
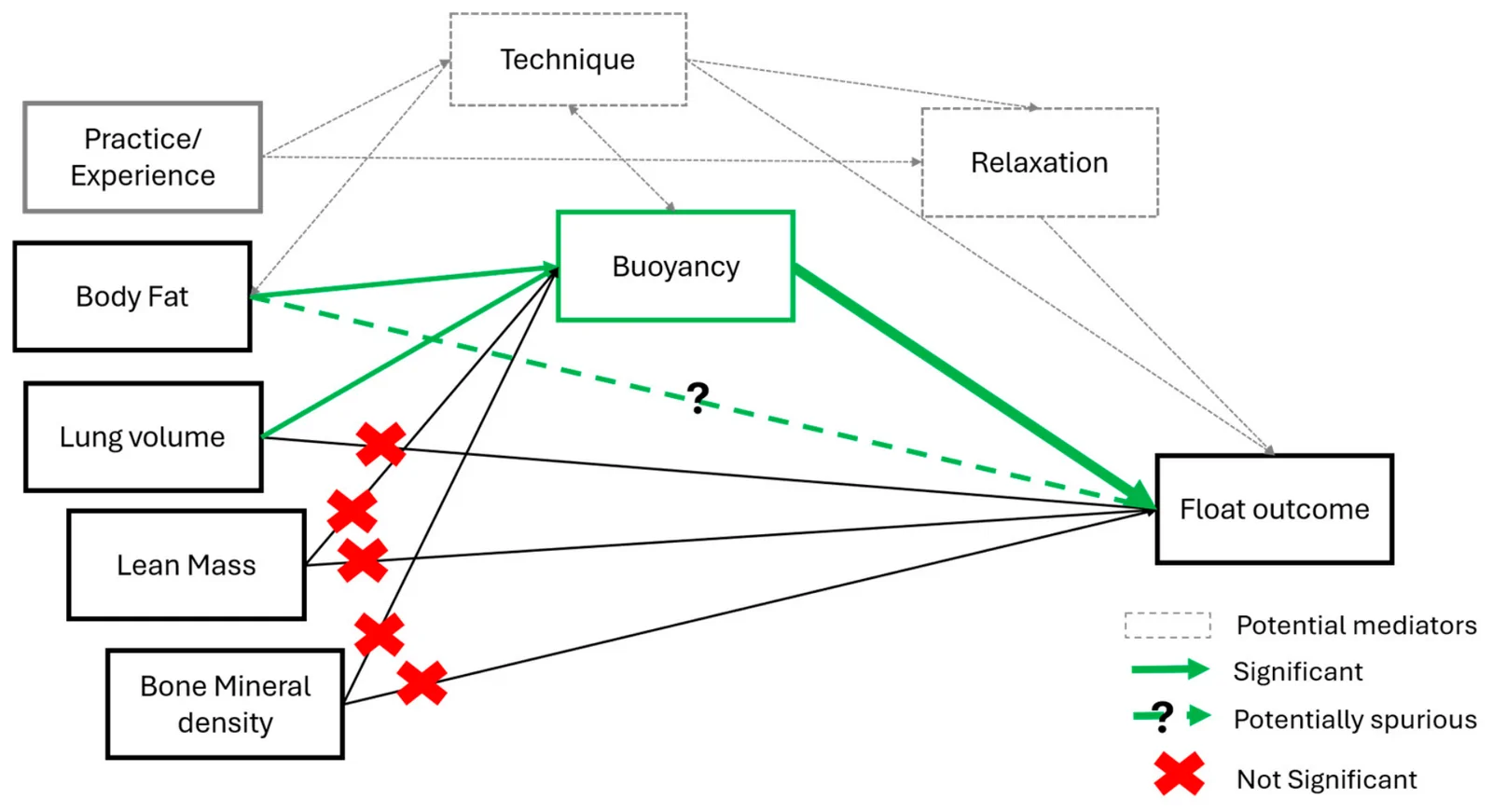
Potential mediators of float outcome. The green dashed line with a question mark indicates a statistically significant direct influence of body fat on float outcome, but has no mechanistic basis; the grey dashed lines indicate potential mechanisms not included in the statistical model which, if included, may remove the direct effect of body fat on float outcome.

In terms of limitations of the work, buoyancy negatively correlated with the effort needed to float (float outcome) and significantly mediated the relationship between body fat, lung volume and float outcome. However, mediation analysis of body composition covariates indicated body fat also had a direct effect on the effort required to float. This apparent direct effect likely reflects unmeasured variables not included in the mediation analysis, such as the technique required to float and capability to relax while immersed (Figure 6). Future analyses should include these factors; doing so may help to account for a greater proportion of the variability in the data, and remove the spurious direct effect that body fat percentage currently appears to have on float outcomes in these statistical models.

Relaxation appeared central to reducing float effort; those reporting lower perceived exertion were more often rated as relaxed, likely because they needed less movement to stay afloat. Relaxation was assessed only subjectively and inferred indirectly from minimal heart-rate changes, as no validated objective measures were available.

Measurement of resting heart rate could provide a useful baseline against which to interpret heart rate during floating and estimate the relative physiological effort required. However, owing to high levels of anxiety among participants prior to water entry, it was not possible to obtain true resting heart rate values. Consequently, the use of these elevated pre-test measurements may have underestimated the effort associated with floating. For pragmatic reasons, heart rate was instead recorded following completion of the practice session, during a brief rest period before the 2 min float.

To allow free floating behaviour, breathing volumes and oxygen cost were not measured. The use of a facemask and mouthpiece would make it challenging to accurately assess the position of the airway in relation to the water surface, and tethering individuals to a fixed position could increase the activity requirement to stay afloat. Instead, participants were advised to take steady tidal breaths and use the minimum effort they needed to keep their airway above the water. The effort to do this was estimated from heart rate and RPE, though both may have been influenced by anxiety, which can elevate heart rate independent of exertion [51]. RPE is also inherently subjective.

The floating technique assessment tool was still under development and unvalidated, so it was excluded from mediation analyses. Nonetheless, technique clearly affected the effort required, underscoring the need for validated, objective tools to assess floating technique in future research.

## 5. Conclusions

This study demonstrates that buoyancy and floating outcome are primarily influenced by body composition, particularly body fat and the volume of air in the lungs, rather than bone mineral density (BMD). Therefore, adults from African, Caribbean and Asian communities can float, and previously held beliefs do not appear relevant for today’s communities.

Importantly, although some participants had low levels of buoyancy, most participants were able to achieve a two-minute float following instruction and practice. These findings show that it is possible to learn the techniques to become competent at floating. This supports the integration of widely deployed drowning prevention campaigns with co-produced opportunities for float practice as a critical pathway to reducing disparities in aquatic activity and drowning.

## Figures and Tables

**Table 1:** Self-reported ethnicity of participants. *n* = 96.

Ethnicity	Number (Percentage)
African American	2 (2.1)
African Caribbean	3 (3.1)
Asian	1 (1.0)
Bengali	1 (1.0)
Black African	15 (15.7)
Black British	11 (11.5)
Black Caribbean	17 (17.7)
Chinese	4 (4.2)
Fijian	1 (1.0)
Indian	11 (11.5)
Jamaican/Somali	1 (1.0)
Japanese	1 (1.0)
Malaysian	2 (2.1)
Mixed Black	6 (6.3)
Mixed Black & White	3 (3.1)
Mixed Black & White African	1 (1.0)
Mixed Caribbean Black & White	7 (7.3)
Mixed Pakistani & White	1 (1.0)
Nigerian	2 (2.1)
Pakistani	2 (2.1)
Thai	3 (3.1)
Vietnamese	1 (1.0)

**Table 2:** Mean (standard deviation) and range of participants’ characteristics. *n* = 96.

		Men	Range	Women	Range
	N=	43		53	
	Age (years)	33 (11)	19–57	41 (12)	19–60
	Height (m)	1.775 (0.073)	1.636–1.967	1.622 (0.072)	1.444–1.748
	Mass (kg)	81.6 (15.0)	48.1–115.7	76.9 (20.4)	47.2–146.3
	BMI (kg.m^−2^)	25.8 (4.0)	16.7–35.3	29.1 (7.0)	18.7–51.3
Girth	Chest (cm)	98.3 (8.8)	72.3–112.8	93.7 (10.6)	76.5–123.5
	Arm (cm)	32.5 (4.1)	23.5–42.9	32.0 (5.4)	22.8–45.0
	Waist (cm)	86.3 (9.8)	60.8–105.2	85.8 (13.9)	63.2–123.5
	Hip (cm)	97.1 (9.8)	77.3–115.2	106.0 (14.1)	83.7–151.8
	Thigh (cm)	55.9 (6.8)	42.4–72.6	60.1 (9.2)	45.1–83.4
Body composition	Waist to hip ratio	0.89 (0.08)	0.78–1.05	0.81 (0.06)	0.69–0.92
	Body fat (%, DXA)	24.89 (5.28)	16.02–35.96	39.01 (7.84)	22.93–57.80
	Body fat (%, RFE)	22.4 (4.7)	9.4–31.8	37.3 (6.0)	23.6–48.7
	Body fat (%, BIA)	20.5 (5.5)	10.1–35.3	36.0 (10.6)	17.2–59.6
	Hip BMD (g.cm^−2^)	1.0 (0.2)	0.7–1.5	0.9 (0.2)	0.5–1.3
	Spine BMD (g.cm^−2^)	1.1 (0.2)	0.8–1.4	1.1 (0.1)	0.8–1.4
	Lean Mass (kg, DXA)	59.95 (9.43)	37.70–85.41	45.18 (7.83)	29.69–66.21
	Buoyancy (N)	7.9 (12.5)	−16.5–30.6	20.5 (13.1)	−4.8–63.7
Lung Volume	FVC (L)	4.8 (1.2)	2.3–7.8	3.23 (0.8)	1.46–5.50

BMI, Body Mass Index; DXA, Dual-energy X-ray; RFE, Relative fat mass equation [37]; BIA, Bio-electrical Impedance; BMD, Bone mineral density; FVC, Forced vital capacity.

**Table 3:** Water-based activity undertaken by participants at the time of the testing.

Activity	Number (Percentage)
No water-based activity	2 (2.10)
No water-based activity but would like to learn	30 (31.25)
Learning to swim	9 (9.37)
Infrequently swim for recreation	31 (32.29)
Swim once a week	4 (4.16)
Swim 2+ times a week	7 (7.29)
Swim for fitness or as part of a swimming club	7 (7.92)
Teach/Coach swimming or am a Lifeguard	5 (5.20)
No information given	1 (1.04)

**Table 4:** Floating positions adopted in the final 30 s of the float of a two min float. In all positions, the legs can be close to the surface or deeper. Some participants adopted multiple floating positions or their position was not noted; float position totals do not sum to *n* = 96.

Short Name	Armchair	Pencil	Scarecrow	Starfish	Torpedo
Description	Seated, hips flexed, knees close to the surface of the water, arms relaxed	Legs and arms extended and abducted, arms extended above head	Legs extended and together, arms adducted and in line with the shoulders	Legs and arms extended and adducted	Legs and arms extended and abducted
Representations					
Plan view	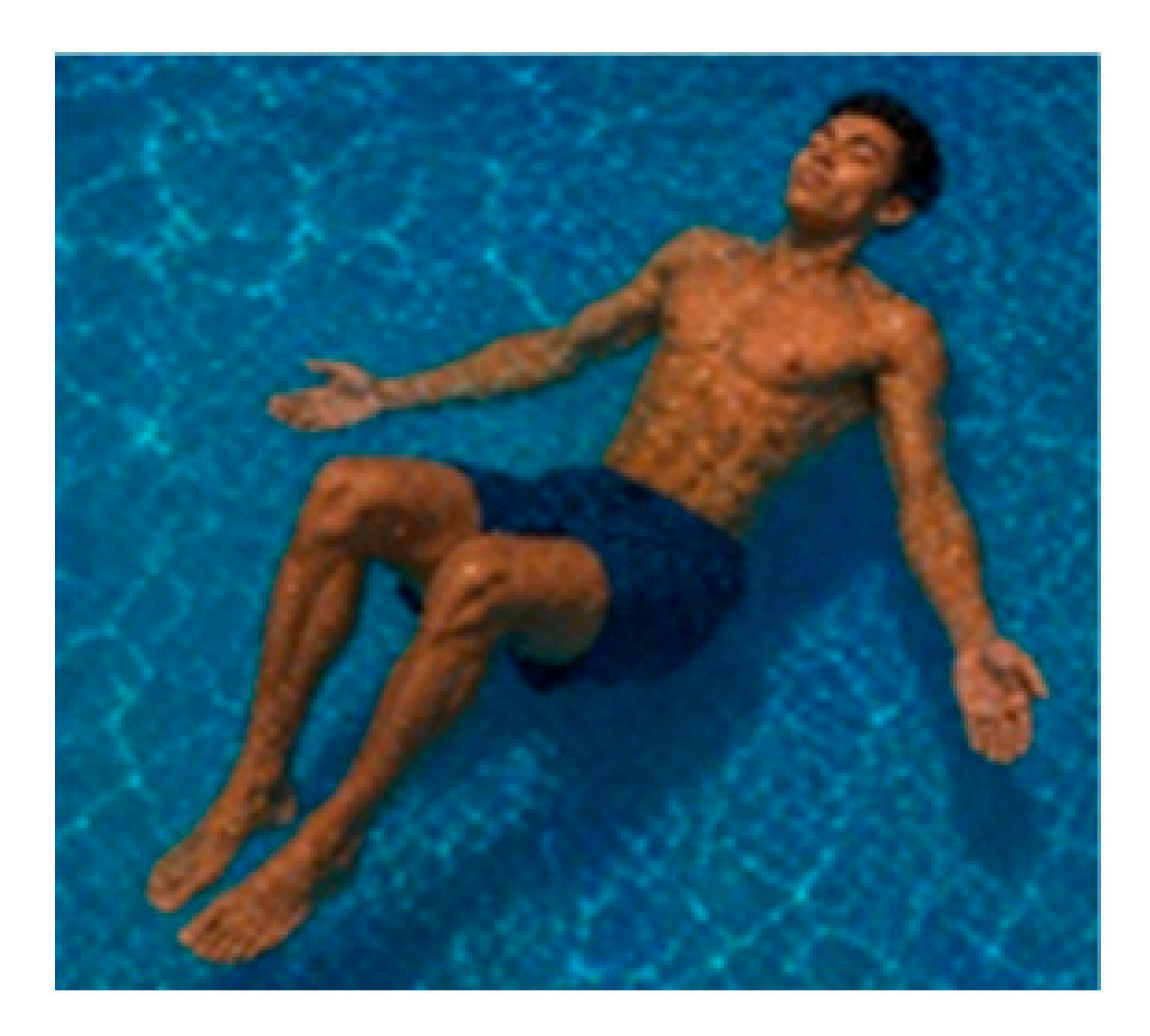	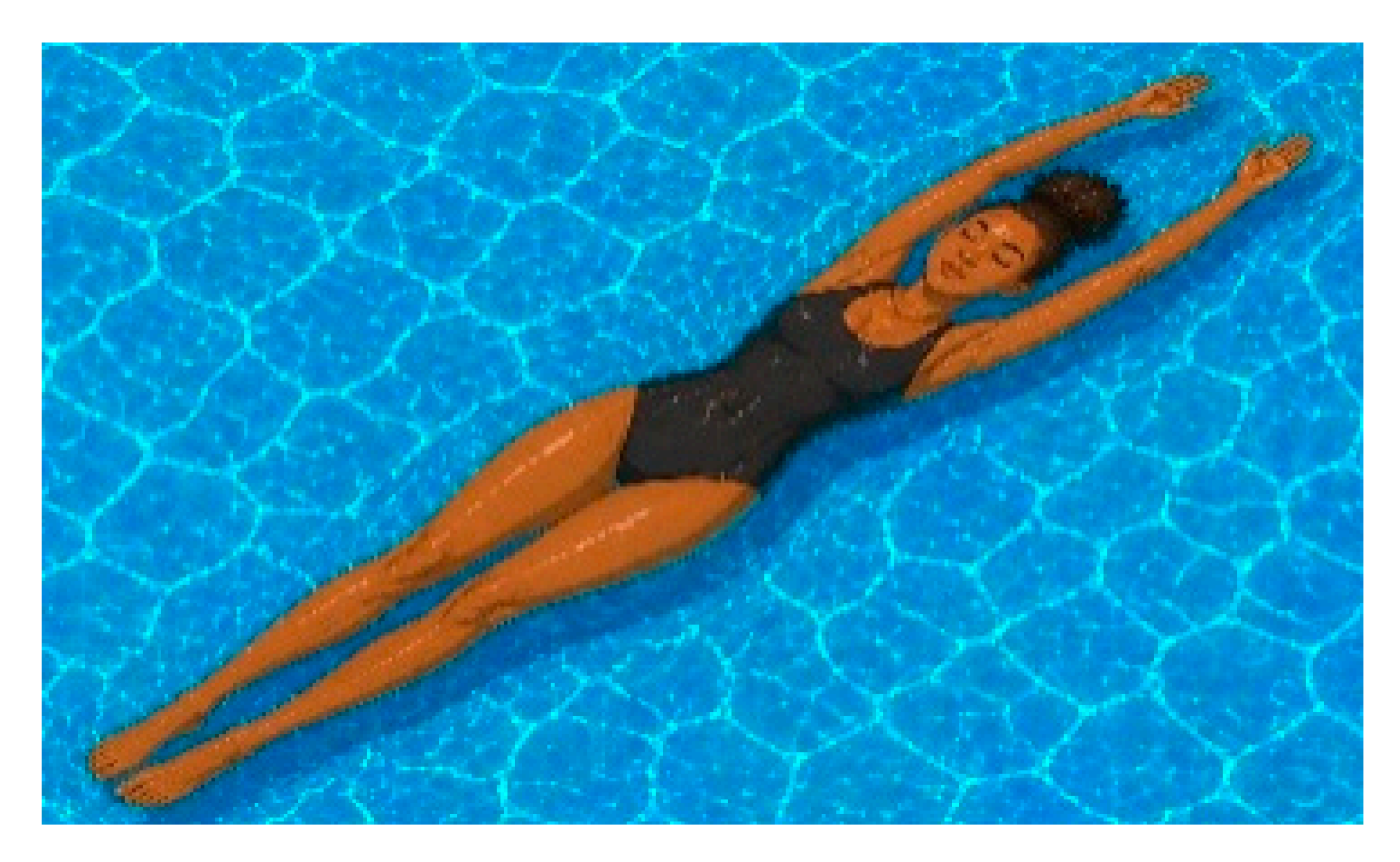	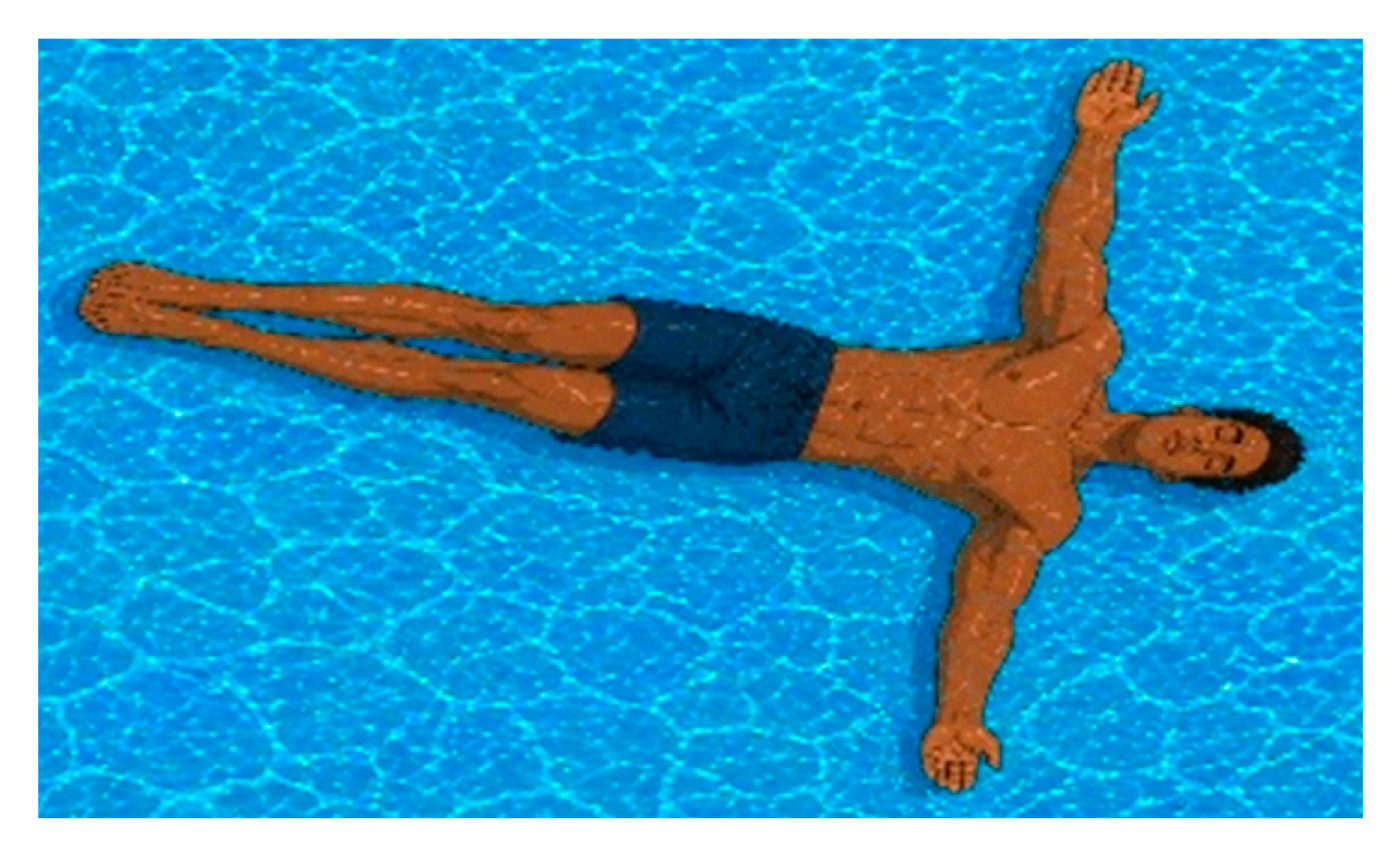	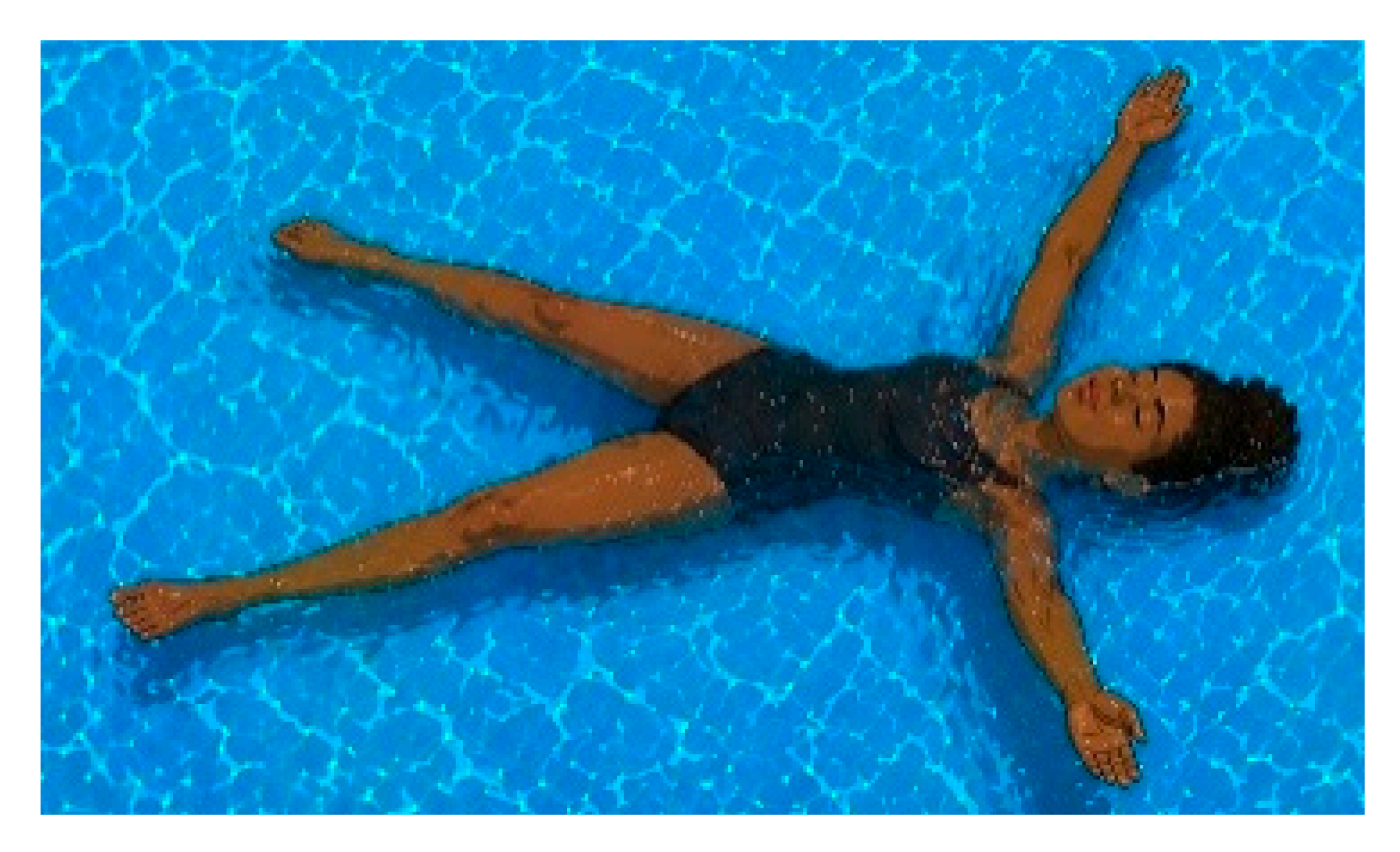	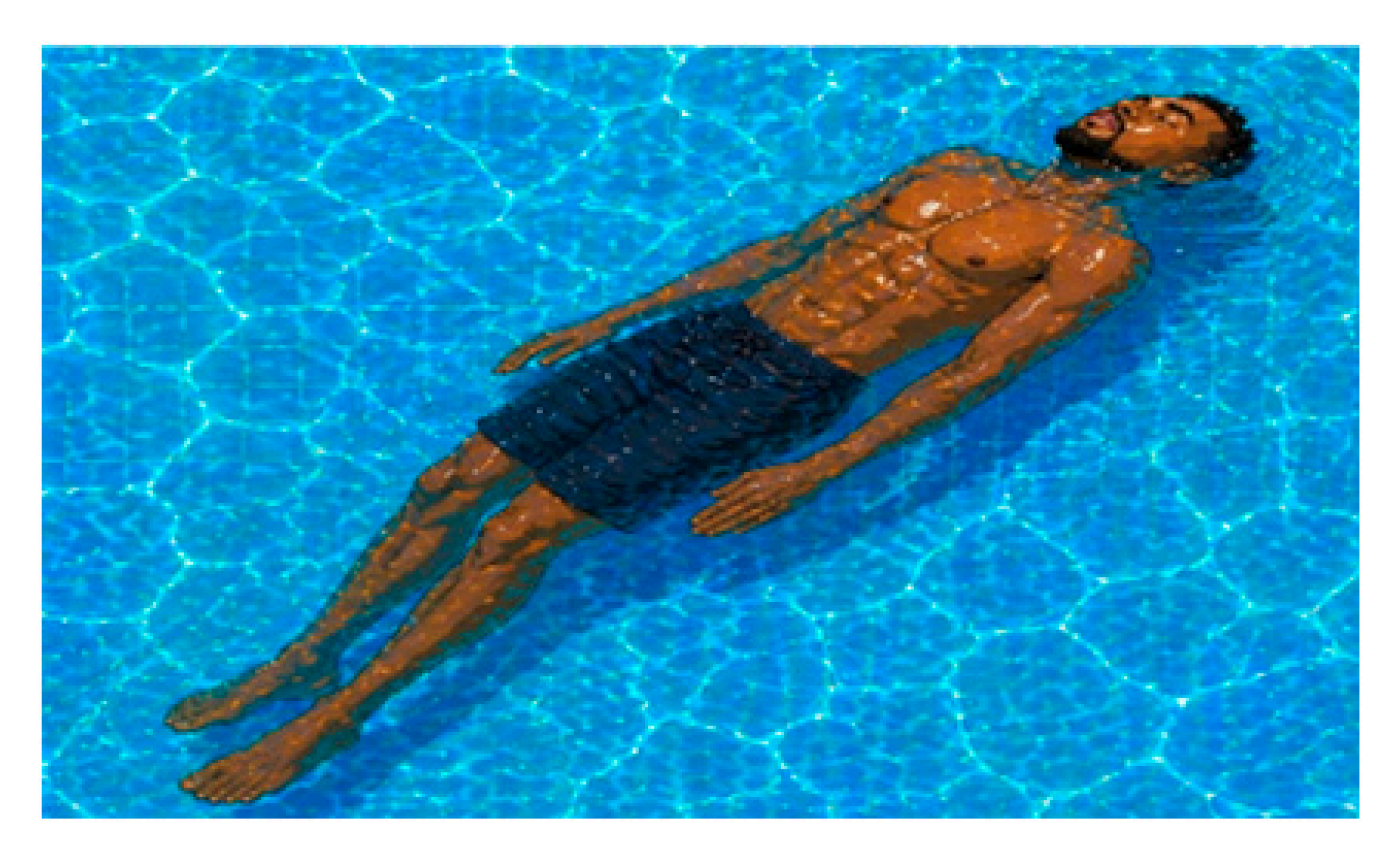
Side view	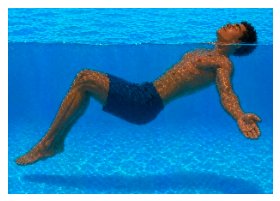	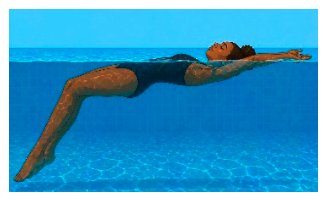	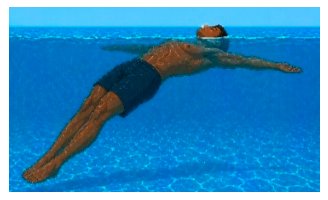	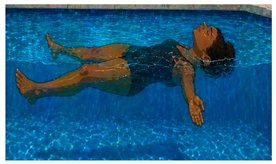	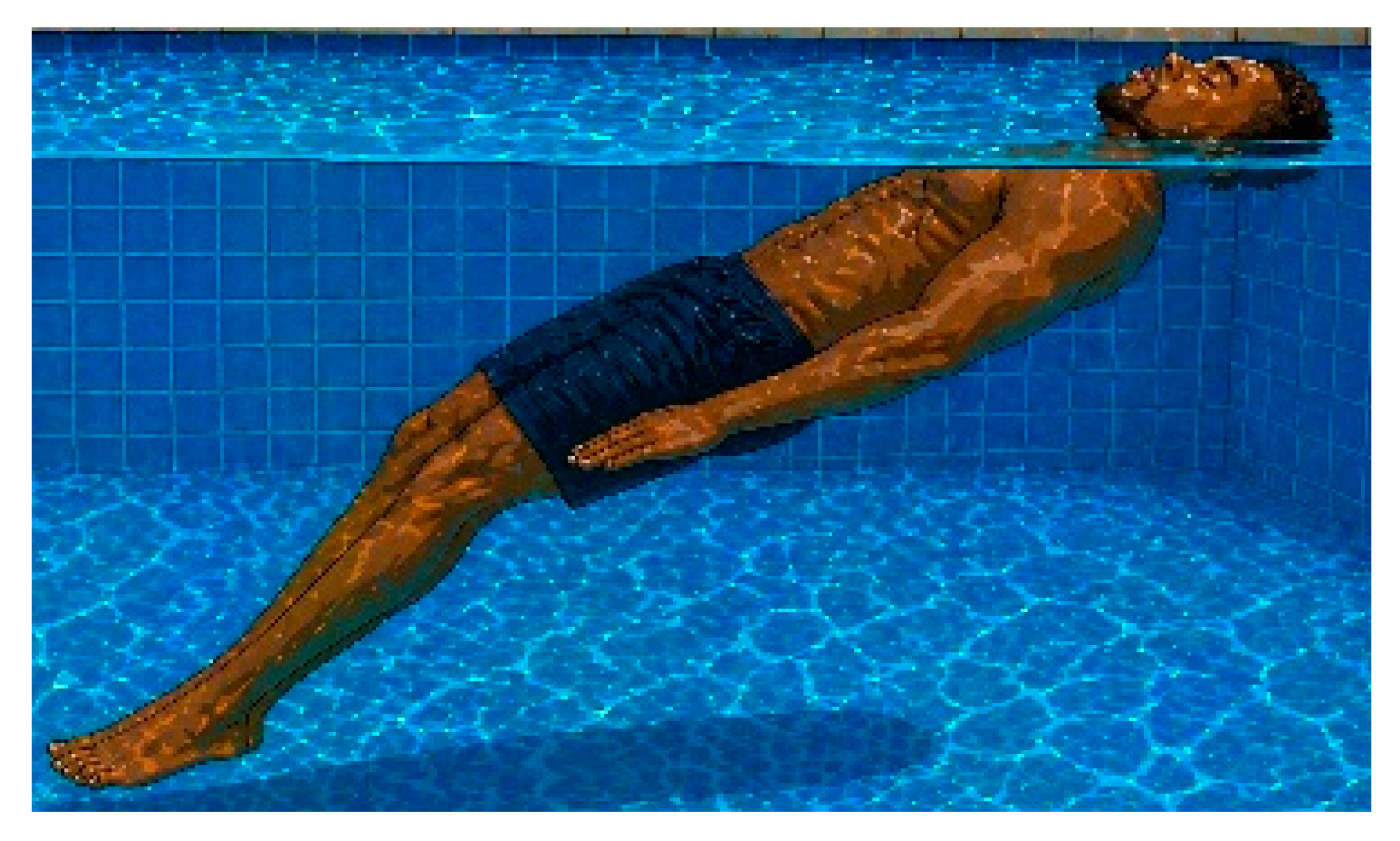
Passive (inactive) N; m/f BMI (kg/m^2^) BF(DXA) (%) BMD (g/cm^2^) Buoyancy (N) Trunk angle (°)	7; 0/7 29.3 (24.8–33.1) 40.3 (25.8–46.3) 1.23 (1.15–1.30) 21.6 (13.1–31.6) 38.9 (19.1–54.5)	4; 0/4 36.2 (26.6–45.1) 43.1 (37.6–47.7) 1.29(1.18–1.38) 33.8 (25.2–51.22) 36.8 (19.9–76.8)	9; 3/6 29.4 (21.9–45.3) 36.2 (26.2–49.5) 1.23(1.06–1.34) 19.7 (1.3–36.1) 50.0 (13.9–82.9)	36; 9/27 28.6 (18.7–45.1) 36.9 (20.1–50.9) 1.2 (0.99–1.39) 21.5 (−2.1–51.22) 39.7 (10.1–84.8)	7; 3/4 30.3 (22.1–51.3) 24.3 (20.5–27.7) 1.17 (0.97–1.28) 24.0 (8.7–63.7) 60.5 (22.83–82.6)
Active N; m/f BMI (kg/m^2^) BF (%) BMD (g.cm^2^) Buoyancy (N) Trunk angle (°)	5; 4/1 25.3 (21.0–28.1) 28.0 (16.2–42.9) 1.29(1.06–1.42) 5.2 (−9.1–19.7) 58.0 (49.1–74.7)	0	8; 4/4 22.9 (17.4–26.4) 26.4(16.0–31.8) 1.23(1.02–1.49) 2.7 (−16.5–18.8) 43.7 (16.6–63.9)	12; 10/2 26.6 (20.8–33.3) 24.9 (19.6–34.5) 1.3 (1.0–1.4) 6 (−10.0–28.7) 42.1 (22.6–59.9)	6; 5/1 26.2 (20.3–31.7) 24.3 (20.5–27.7) 1.34 (1.14–1.47) 3.9 (−9.6–24.8) 43.6 (12.3–66.5)

Representative images of the different postures adopted were recreated using ChatGTP 5.5. based on images recorded during data collection.

**Table 5:** Floating competence, body tension, head position, arm and leg activity assessed during the last 30 s of float. *n* (%).

Float Outcome	Float Duration (s)	Airway Position Number of Submersions in Final 30 s of Float					Head Position			Relaxation		
		Never	Occasionally (1–2)	Often (3–5)	Frequently (5+)	Constantly	Back	Neutral	Forward	Relaxed	Neutral	Tense
Floats with no effort (*n* = 50)	120 ± 0	43 (45)	5 (5)				33 (34)	15 (16)		20 (21)	20 (21)	8 (8)
Floats with little effort (*n* = 17)	120 ± 0	15 (16)		2 (2)			12 (13)	4 (4)	1 (1)	1 (1)	14 (15)	2 (2)
Floats with some effort (*n* = 13)	117 ± 9	13 (14)	1 (1)	1 (1)			12 (13)	3 (3)			3 (3)	12 (13)
Floats with lots of effort (*n* = 9)	120 ± 0	4 (4)	4 (4)	1 (1)			9 (9)					9 (9)
Could not float (*n* = 7)	21 ± 25	1 (1) *		1 (1)		5 (5)	7 (7)				3 (3)	4 (4)

* The individual whose airway was never submerged was classified as “could not float” because the lifeguards had constant contact with them.

**Table 6:** Mean and range of anthropometric measures and confidence and difficulty ratings of men who did not float (*n* = 7) compared to men who did float (*n* = 36). Effect sizes are presented as Hedges’ *g*. Non-parametric data analysis, noted in italics, as the median (range) and *r* as the effect size.

	Did Not Float	Floated	*p*	Effect Size
*n*	7	36		
Height (m)	1.72 (1.67–1.80)	1.78 (1.63–1.96)	**0.010**	**0.93**
Mass (kg)	65.7 (50.1–82.2)	84.7 (48.05–115.70)	**0.001**	**1.54**
BMI (kg.m^−2^)	22.2 (16.7–28.1)	26.4 (17.4–35.3)	**0.006**	**1.12**
BF_(DXA)_ (%)	21.5 (16.3–30.1)	25.5 (16.0–36.0)	0.063	0.79
BF_(RFE)_ (%)	19.7 (12.6–25.9)	22.9 (9.4–31.8)	0.084	0.68
BF_(BIA)_ (%)	19.5 (10.1–28.7)	21.56 (10.8–55.5)	0.535	0.27
Lean Mass_(DXA)_ (kg)	51.1 (41.6–60.1)	61.7 (37.7–85.4)	**0.005**	**1.38**
BMD (g.cm^−2^) whole body	1.22 (1.03–1.42)	1.28 (1.02–1.49)	0.142	0.57
BMD (g.cm^−2^) hip	0.91 (0.76–1.13)	1.02 (0.65–1.53)	0.071	0.79
BMD (g.cm^−2^) spine	1.01 (0.77–1.13)	1.11 (0.76–1.42)	0.175	0.60
Buoyancy (N)	1.0 (−7.7–15.0)	9.6 (−16.5–30.6)	0.108	0.77
Forced Vital Capacity (FVC, L)	3.98 (3.74–4.20)	4.95 (2.27–7.75)	<0.001	1.07
Heart rate (bpm, before float)	84 (59–151)	84 (56–125)	0.769	0.02
Heart rate (bpm, float end)	114 (82–158)	87 (67–123)	** <0.001 **	** 0.71 **
RPE (after float)	4 (2–7)	3 (0–6)	** 0.048 **	** −0.73 **
Float confidence (before)	2 (2–4)	3 (1–5)	0.754	0.01
Float confidence (after)	4 (3–4)	4 (2–5)	0.139	0.26
Float difficulty (before)	3 (1–4)	3 (1–5)	0.932	0.02
Float difficulty (after)	3 (3–4)	4 (2–5)	0.220	0.20
Attended swimming lessons (*n*)	Yes 5, No 2	Yes 31, No 5	0.318	0.40
Self-identified swimming capability (1–10)	5 (1–8)	5 (1–10)	0.572	0.09

Bold *p* values and effect sizes note *p* < 0.05. BMI, Body Mass Index; DXA, Dual-energy X-ray; RFE, Relative fat mass equation [37]; BIA, Bio-electrical Impedance Analysis; BMD, Bone mineral density; FVC, Forced vital capacity; RPE, Rate of perceived exertion (0–10 scale), Float confidence (1–5 scale), Float difficulty (1–5 scale).

**Table 7:** Correlation matrix showing the relationships between floating characteristics and body composition measures. *r* values (*p* values). Bold text indicates correlations (*r* > 0.6 or <−0.6).

			Body Fat (%)					Lean Mass		Bone Mineral Density (g.cm^−2^)			
	Float Outcome	Buoyancy (N)	DXA	RFE	BIA	Android	Gynoid	Dry Lean Mass (BIA) (%)	Lean Mass (DXA) (kg)	Whole Body	Hip	Spine	FVC (L)
Float outcome		**0.681** **(<0.001)**	**0.65** **(<0.001)**	**0.613** **(<0.001)**	0.505 (<0.001)	**0.607** **(<0.001)**	**0.676** **(<0.001)**	−0.274 (0.006)	−0.191 (0.061)	−0.274 (0.006)	−0.145 (0.158)	−0.010 (0.923)	−0.116 (0.258)
Buoyancy (N)	**0.681** **(<0.001**)		**0.763** **(<0.001)**	**0.653** **(<0.001)**	0.571 (<0.001)	**0.750** **(<0.001)**	**0.732** **(<0.001)**	−0.331 (0.001)	−0.143 (0.163)	−0.289 (0.004)	−0.172 (0.094)	0.070 (0.494)	−0.114 (0.267)
Trunk angle (°)	−0.169 (0.105)	−0.380 (<0.001)	−0.480 (<0.001)	−0.414 (<0.001)	−0.317 (0.002)	−0.431 (<0.000)	−0.456 (<0.001)	0.245 (0.018)	0.207 (0.047)	0.144 (0.170)	0.081 (0.444)	0.093 (0.378)	0.327 (0.002)
Relaxation–tension	0.568 (<0.001)	**0.629** **(<0.001)**	**0.608** **(<0.001)**	0.569 (<0.001)	0.446 (<0.001)	0.564 (<0.001)	0.572 (<0.001)	−0.239 (0.018)	−0.095 (<0.001)	−0.251 (0.013)	−0.062 (0.545)	−0.05 (0.585)	−0.150 (0.142)
Post float RPE	0.534 (<0.001)	−0.467 (<0.001)	−0.463 (<0.001)	−0.404 (<0.001)	−0.393 (<0.001)	−0.433 (<0.001)	−0.469 (<0.001)	0.260 (0.061)	0.139 (0.006)	0.244 (0.006)	0.053 (0.158)	0.057 (0.922)	0.116 (0.258)
Arm movement frequency	**−0.799** **(<0.001)**	−0.567 (<0.001)	−0.523 (<0.001)	−0.461 (<0.001)	−0.393 (<0.001)	−0.489 (<0.001)	−0.539 (<0.001)	0.214 (0.036)	0.111 (0.279)	0.304 (0.002)	0.197 (0.054)	0.106 (0.300)	0.079 (0.441)
Arms movement intensity	**−0.791** **(<0.001)**	**−0.611** **(<0.001)**	−0.566 (<0.001)	−0.516 (<0.001)	−0.459 (<0.001)	−0.524 (<0.001)	**−0.603** **(<0.001)**	0.301 (0.003)	0.188 (0.066)	0.383 (0.001)	0.290 (0.004)	0.128 (0.210)	0.110 (0.281)
Arms work/rest ratio	**−0.805** **(<0.001)**	−0.596 (<0.001)	−0.543 (<0.001)	−0.497 (<0.001)	−0.462 (<0.001)	−0.516 (<0.001)	−0.574 (<0.001)	0.257 (0.011)	0.109 (0.290)	0.258 (0.010)	0.193 (0.060)	0.058 (0.570)	0.066 (0.522)
Legs movement frequency	−0.412 (<0.001)	−0.424 (<0.001)	−0.336 (<0.001)	−0.251 (0.0134)	−0.265 (0.008)	−0.331 (<0.001)	−0.351 (0.001)	0.221 (0.030)	0.236 (0.020)	0.280 (0.005)	0.160 (0.120)	0.099 (0.334)	0.051 (0.616)
Legs movement intensity	−0.441 (<0.001)	−0.449 (<0.001)	−0.351 (0.001)	−0.265 (0.009)	−0.263 (0.009)	−0.338 (<0.001)	−0.372 (<0.001)	0.196 (0.054)	0.263 (0.009)	0.330 (0.001)	0.188 (0.066)	0.126 (0.221)	0.058 (0.572)
Legs work/rest ratio	−0.382 (0.001)	−0.392 (<0.001)	−0.299 (0.003)	−0.228 (0.024)	−0.258 (0.011)	−0.303 (<0.001)	−0.308 (0.002)	0.184 (0.071)	0.159 (0.120)	0.195 (0.056)	0.107 (0.298)	0.054 (0.595)	−0.001 (0.993)

DXA, Dual-energy X-ray; RFE, Relative fat mass equation [37]; BIA, Bio-electrical Impedance Analysis; BMD, Bone mineral density; FVC, Forced vital capacity; RPE, Rate of perceived exertion (0–10 scale).

**Table 8:** Correlation matrix showing the relationships between floating characteristics and more accessible body composition measures. *r* values (*p* values). Bold text indicates correlations (*r* > 0.6 or <−0.6).

					Girths						
		Height (cm)	Mass (kg)	BMI (kg.m^−2^)	Arm (cm)	Chest (cm)	Waist (cm)	Hip (cm)	Thigh (cm)	Post Float RPE	Relaxation–Tension
	Float outcome	−0.240 (0.018)	0.247 (0.014)	0.394 (<0.001)	0.208 (0.041)	0.194 (0.058)	0.335 (<0.001)	0.399 (<0.001)	0.332 (<0.001)	0.534 (<0.001)	0.568 (<0.001)
	Buoyancy (N)	−0.218 (0.032)	0.434 (<0.001)	**0.601 (<0.001)**	0.391 (<0.001)	0.385 (<0.001)	0.542 (<0.001)	**0.632 (<0.001)**	0.481 (<0.001)	−0.467 (<0.001)	**0.629 (<0.001)**
	Trunk angle (°)	0.268 (0.009)	−0.154 (0.141)	−0.335 (0.001)	−0.186 (0.074)	−0.080 (0.444)	−0.204 (0.051)	−0.346 (<0.001)	−0.322 (0.002)	0.289 (0.005)	−0.482 (<0.001)
	Post float RPE	0.195 (0.057)	−0.180 (0.077)	−0.291 (0.003)	−0.156 (0.128)	−0.151 (0.140)	−0.243 (0.016)	−0.272 (0.007)	−0.252 (0.013)		−0.529 (<0.001)
Arms	Movement frequency	0.109 (0.286)	−0.254 (0.012)	−0.339 (<0.001)	−0.191 (0.061)	−0.192 (0.060)	−0.331 (<0.001)	−0.378 (<0.001)	−0.293 (0.003)	0.390 (<0.001)	−0.529 (<0.001)
	Movement Intensity	0.175 (0.088)	−0.203 (0.046)	−0.315 (0.002)	−0.153 (0.135)	−0.128 (0.212)	−0.301 (0.002)	−0.416 (<0.001)	−0.282 (0.005)	0.472 (<0.001)	−0.595 (<0.001)
	Work/rest ratio	0.149 (0.147)	−0.270 (0.007)	−0.370 (<0.001)	−0.227 (0.026)	−0.224 (0.027)	−0.343 (<0.001)	−0.414 (0.001)	−0.313 (0.002)	0.437 (<0.001)	−0.514 (<0.001)
Legs	Movement frequency	0.191 (0.061)	−0.024 (0.810)	−0.145 (0.158)	−0.020 (0.844)	−0.034 (0.737)	−0.100 (0.331)	−0.139 (0.174)	0.011 (0.909)	0.184 (0.072)	−0.304 (0.003)
	Movement Intensity	0.189 (0.063)	−0.003 (0.973)	−0.124 (0.227)	0.015 (0.884)	−0.015 (0.880)	−0.096 (0.347)	−0.149 (0.144)	0.015 (0.877)	0.213 (0.036)	−0.330 (<0.001)
	Work/rest ratio	0.143 (0.163)	−0.068 (0.504)	−0.164 (0.109)	−0.067 (0.511)	−0.072 (0.480)	−0.136 (0.184)	−0.141 (0.168)	−0.018 (0.860)	0.155 (0.130)	−0.260 (0.010)

BMI, Body Mass Index; RPE, Rate of perceived exertion (0–10 scale).

**Table 9:** Characteristics of participants who were active or passive during the two min float. Mean (SD) with Hedges *g* effect sizes unless in italics, which indicates median (range) with *r* effect sizes.

	Active Float	Passive Float	*p*	Effect Size
*n*	30; 8 f, 22 m	59; 45 f, 14 m		
Float duration (s)	118 (6)	120 (0)	0.167	−0.117
Height (m)	172.4 (9.9)	166.9 (10.9)	**0.022**	**−0.095**
Mass (kg)	76.1 (17.3)	82.1 (18.6)	0.145	0.281
BMI (kg.m^−2^)	25.3 (3.8)	29.5 (6.5)	**0.002**	**0.377**
Arm Circumference (cm)	31.4 (4.8)	33.0 (4.8)	0.148	0.133
Chest Circumference (cm)	94.9 (10.5)	96. 9 (9.8)	0.384	0.317
Waist Circumference (cm)	80.9 (10.7)	89.5 (12.1)	**0.001**	**0.513**
Hip Circumference (cm)	95.6 (9.3)	106.4 (13.5)	**<0.001**	**0.300**
Thigh Circumference (cm)	55.6 (7.2)	60.4 (8.3)	**0.008**	**0.293**
Waist to hip ratio	0.86 (0.1)	0.83 (0.1)	0.881	0.346
BF_(DXA)_ (%)	25.8 (5.7)	37.5 (8.6)	**<0.001**	**0.364**
BF_(RFE)_ (%)	24.0 (5.5)	35.3 (7.9)	**<0.001**	**0.598**
BF_(BIA)_ (%)	22.1 (6.6)	34.4 (11.1)	**<0.001**	**0.412**
Lean Mass_(DXA)_ (kg)	55.7 (13.1)	49.9 (10.4)	**0.025**	**0.006**
BMD (g.cm^−2^) whole body	1.3 (0.1)	1.2 (0.1)	**0.003**	**0.118**
BMD (g.cm^−2^) hip	1.0 (0.2)	0.9 (0.2)	**0.024**	**0.180**
BMD (g.cm^−2^) spine	1.1 (0.2)	1.1 (0.1)	0.577	0.131
Buoyancy (N)	4.5 (11.0)	21.8 (11.7)	**<0.001**	**0.301**
Forced Vital Capacity (L)	4.3 (1.1)	3.7 (1.4)	**0.025**	**−0.063**
Trunk angle (°)	45.1 (16.1)	43.6 (21.9)	0.713	0.027
Heart rate (before float, bpm)	84 (59–151)	84 (56–125)	0.769	0.02
Heart rate (after float, bpm)	114 (82–158)	87 (67–123)	** <0.001 **	** 0.71 **
RPE (before float)	1 (0–5)	1 (0–6)	0.124	−0.125
RPE (after float)	2 (0–7)	2 (0–6)	0.611	−0.029
Float confidence (before practice)	4 (1–5)	3 (1–5)	0.662	0.031
Float confidence (after float)	4 (3–5)	4 (2–5)	0.906	−0.007
Float difficulty (before practice)	3 (1–5)	3 (1–5)	0.118	−0.160
Float difficulty (after float)	4 (2–5)	4 (2–5)	** <0.001 **	** −0.385 **

Bold *p* values and effect sizes note *p* < 0.05. BMI, Body Mass Index; DXA, Dual-energy X-ray; RFE, Relative fat mass equation [37]; BIA, Bio-electrical Impedance Analysis; BMD, Bone mineral density; FVC, Forced vital capacity; RPE, Rate of perceived exertion (0–10 scale).

## Data Availability

The anonymised data collected in the study are openly available in the University of Portsmouth online data repository at https://doi.org/10.17029/9676a5e3-078b-45a1-a35e-f1d47b2531d3.

## Supplementary Materials

The following supporting information can be downloaded at: https://www.mdpi.com/article/10.3390/ijerph23080990/s1, Supplementary S1: People of African, Caribbean and Asian heritage “Find Your Float”: a Cross-Sectional Study [37]. Table S1.1: Float techniques descriptions; Table S1.1b Interrater reliability on a subset of 30 s Float data (*n* = 12); Table S1.2: Inter- and intrarater reliability of accessible body measurements; Table S1.2a: Interrater reliability of height (cm) measurements recorded from 3 participants by each of the 7 researchers, ICC = 0.995; Table S1.2b: Interrater reliability of mass (kg) measurements recorded from 3 participants by each of the 7 researchers, ICC = 1.00; Table S1.2c: Interrater reliability of arm girth (cm) measurements recorded from 3 participants by each of the 7 researchers, ICC = 0.993; Table S1.2d: Interrater reliability of chest girth (cm) measurements recorded from 3 participants by each of the 7 researchers, ICC = 0.9896; Table S1.2e: Interrater reliability of waist girth (cm) measurements recorded from 3 participants by each of the 7 researchers, ICC = 0.984; Table S1.2f: Interrater reliability of hip girth (cm) measurements recorded from 3 participants by each of the 7 researchers, ICC = 0.9979; Table S1.2g: Interrater reliability of thigh girth (cm) measurements rec-orded from 3 participants by each of the 7 researchers, ICC = 0.9945; Table S1.2h: Interrater relia-bility of forced vital capacity (L) measurements recorded from 3 participants by each of the 7 re-searchers, ICC = 0.7927; Table S2.1. Frequency (percentages) of arm movements during the last 30 s of a 2 min float separated by float outcome; Table S2.2. Frequency (percentages) of leg action during the last 30 s of a 2 min float separated by float outcome; Figure S1.1 Frequency distributions of male (purple bars *n* = 43) and female (orange bars *n* = 53) participants for (A) age (years), (B) height (m), (C) mass (kg), (D) BMI (kg.m^−2^), (E) arm (cm), (F) chest, (cm), (G) waist (cm), (H) hip (cm) and (I) thigh (cm) girths, (J) waist-to-hip ratio, and (K) relative fat equation (%) ^1^; Figure S1.2 Frequency distributions of male (purple bars *n* = 43) and female (orange bars *n* = 53) participants for (A) DXA derived body fat (%), (B) lean body mass derived from DXA (kg), (C) whole body bone mineral density (g.cm^−2^), (D) hip bone mineral density (g.cm^−2^), (E) spine bone mineral density (g.cm^−2^); Figure S1.3. Responses to floating in participants who floated actively or passively. A. Heart rate at the start of the float and end of the float, B. rate of perceived exertion, C. confidence, D. difficulty before and immediately after the float, and E. the perception of difficulty in response to the question: How did floating compare to how difficult you thought it would be? The width of the violin plots indicates the frequency of votes. Bars indicate significant difference, * *p* < 0.05, ** *p* < 0.01, *** *p* < 0.001, **** *p* < 0.0001; Figure S1.4 Relationship between float outcome and buoyancy; Figure S1.5. Anthropometric and physiologic measurements correlated against buoyancy. A, Height (cm); B, mass (kg); C, Body Mass Index (kg.m^−2^); D, arm circumference (cm); E, chest circumference (cm); F, waist circumference (cm); G, hip circumference (cm); H, thigh circumference (cm); I, waist-to-hip ratio; J, forced vital capacity (L); Figure S1.6. Body composition measurements correlated against Buoyancy. A, Body fat (%) measured from DXA scans; B, body fat (%) measured from Bio-electrical impedance; C, body fat (%) calculated from relative fat equations1; D, lean (%) wet measured by DXA; E, dry lean (%) dry measured by Bio-electrical impedance; F, Android and Gynoid fat (%); G, Android fat (%); H, Gynoid fat (%); I, bone mineral density of the whole body (g.cm^−2^); J, bone mineral density of the spine (g.cm^−2^); K, bone mineral density of the hip (g.cm^−2^). Figure S1.7 Floatation descriptors correlated against buoyancy. A, Airway position; B, head position; C, trunk angle (measured,°); D, arm movement frequency; E, arm movement intensity; F, arm movement work rest intervals; G, leg movement frequency; H, leg movement intensity; I, leg movement work rest intervals; J, tension relaxation; K, passive or active floatation; L, trunk position. Figure S1.8 Anthropometric and physiologic measurements correlated against float outcome. A, Height (cm); B, mass (kg); C, Body Mass Index (kg.m^−2^); D, arm circumference (cm); E, chest circumference (cm); F, waist circumference (cm); G, hip circumference (cm); H, thigh circumference (cm); I, waist-to-hip ratio; J, forced vital capacity (L). Figure S1.9. Body composition measures correlated against Float outcome. A, Body fat (%) measured from DXA scans; B, body fat (%) measured from Bio-electrical impedance; C, body fat (%) calculated from relative fat equations1; D, lean (%) wet measured by DXA; E, dry lean (%) dry measured by Bio-electrical impedance; F, Android and Gynoid fat (%); G, Android fat (%); H, Gynoid fat (%); I, bone mineral density of the whole body (g.cm^−2^); J, bone mineral density of the spine (g.cm^−2^); K, bone mineral density of the hip (g.cm^−2^). Figure S1.10 Floatation descriptors correlated against float outcome. A, Airway position; B, head position; C, trunk angle (measured,°); D, arm movement frequency; E, arm movement intensity; F, arm movement work rest intervals; G, leg movement frequency; H, leg movement intensity; I, leg movement work rest intervals; J, tension relaxation; K, passive or active floatation; L, trunk position. Supplemental Figure S1.11. Limits of agreement for body fat measurement methods. (A) DXA and BIA; (B) DXA and relative fat equation.

## Abbreviations

The following abbreviations are used in this manuscript:

BIA	Bio-electrical Impedance Analysis
BMD	Bone Mineral Density
BSA	Black Swimming Association
CI	Confidence Interval
CSR	Cold Shock Response
DXA	Dual-Energy X-ray Absorptiometry
FVC	Forced Vital Capacity (L)
RNLI	Royal National Lifeboat Institution
RFE	Relative Fat Mass
RPE	Rating of Perceived Exertion
SE	Standard Error
VIF	Variance Inflation Factor
