# Supplementary material for: ‘Find Your Float’: A Public Health Approach to Addressing Drowning Inequities in People of African, Caribbean and Asian Heritage: A Cross-Sectional Study
Source: Int J Environ Res Public Health. 2026 Jul 29;23(8):990. doi: 10.3390/ijerph23080990 (PMC13512758; doi:10.3390/ijerph23080990)
Supplement: Supplementary file 1 [file ijerph-23-00990-s001.zip › ijerph-4404989-supplementary.pdf]

Supplementary S1: People of African, Caribbean and Asian heritage "Find Your Float": a Cross-Sectional Study

Page Number

1. Additional Methods 2
2. Additional Results 8
3. Additional Qualitative Analysis 22

1. Additional Methods

Table S1.1 Float techniques descriptions.

Item	Scale
**Airway position**	0 Constantly submerged<br>1 Frequently submerged (>5 times in 30 s)<br>2 Often submerged (3-5 times in 30 s)<br>3 Occasionally submerged (1-2 times in 30 s)<br>4 Never submerged (in the 30 s period)
**Head /Neck position**	0 Head forwards, looking towards feet<br>1 Head neutral, neck straight<br>2 Head back, neck extended, looking up/backwards
**Trunk position**<br>(Trunk angle in brackets)	0 Horizontal (flat on the surface of the water 0-30°)<br>1 Diagonal (31 - 60°)<br>2 Vertical (61- 90°)
**Tension-Relaxation**	0 Tense, Rigid, Stiff, movement required<br>1 Neutral, some tension/relaxation, some parts of the body were supported by the water, others remained rigid/showed movement<br>2 Relaxed, no obvious signs of muscle tension; adopted a posture where their body and limbs were naturally supported by the water
**Arms:**	
**Frequency of movement**	3 Fast<br>2 Moderate<br>1 Slow<br>0 No movement/very low frequency
**Intensity**	3 High intensity<br>2 Moderate intensity<br>1 Low intensity<br>0 No movement
**Work-rest Pattern**	4 Continuous, without rest<br>3 bouts of activity (activity > rest)<br>2 Intermittent (activity = rest)<br>1 occasional (activity < rest)<br>0 No movement
**Size of movement**	No movement<br>Hands only<br>Hands and forearms<br>Whole arm
**Coordination**	Mirrored (synchronous)<br>Alternating (asynchronous)
**Technique**	No movement<br>Sculling<br>Circles<br>Push down<br>Flutter/flap/sweep<br>Other
**Legs:**	
**Frequency of movement**	3 Fast<br>2 Moderate<br>1 Slow<br>0 No movement/very low frequency

**Intensity**	3 High intensity<br>2 Moderate intensity<br>1 Low intensity<br>0 No movement
**Work-rest Pattern**	4 Continuous, without rest<br>3 bouts of activity (activity > rest)<br>2 Intermittent (activity = rest)<br>1 occasional (activity < rest)<br>0 No movement
**Size of movement**	No movement<br>feet only<br>feet and lower legs<br>Whole leg
**Coordination**	Mirrored (synchronous)<br>Alternating (asynchronous)
**Technique**	No Movement<br>Breaststroke/Egg beater<br>Flick or flutter kick<br>Cycle<br>Other
**Float outcome**	4 Cannot float (unable to maintain the required floating position despite continuous effort or who were unable to remain at the water surface without external assistance)<br>3 Floats with lots of effort (required frequent and substantial movements to remain afloat)<br>2 Floats with some effort (regular but moderate movements of the limbs to maintain the floating position)<br>1 Floats with little effort (occasional or minimal corrective movements were required)<br>0 Floats with no effort (floating maintained without any purposeful movement)
**<u>Float shape</u>**	
**Armchair**	Adopting a seated position in the water
**Pencil**	Legs together with the arms above the head brushing the ears (horizontal only)
**Scarecrow**	Arms straight out to the side and legs together
**Starfish**	Arms and legs spread out
**Torpedo**	Legs together and arms by the sides of the body
**Armchair**	Adopting a seated position in the water

Description of floating shapes: these shapes were performed as passive or active floats, in horizontal, diagonal/angled float or vertical in the water, with the exception of the pencil float, which could only be performed in a horizontal position.

Table S1.1b Interrater reliability on a subset of 30 s Float data (n=12)

	Frequency		Work Rest		Float outcome	
	HM	CE	HM	CE	HM	CE
1	1	1	1	1	2	2
2	2	1	2	2	2	2
3	0	0	0	0	0	0
4	2	1	1	2	2	1
5	0	0	0	0	0	1
6	3	3	3	2	3	3
7	2	2	2	1	2	2
8	3	3	3	2	3	3
9	0	0	0	0	0	0
10	3	3	3	3	3	3
11	3	2	3	2	3	3
12	0	0	0	0	0	0
Weighed Cohen's κ	0.917		0.844		0.943	

Table S1.2 Inter- and intrarater reliability of accessible body measurements

Supplemental Table S1.2a. Interrater reliability of height (cm) measurements recorded from 3 participants by each of the 7 researchers, ICC = 0.995

	Researcher 1	Researcher 2	Researcher 3	Researcher 4	Researcher 5	Researcher 6	Researcher 7
Participant 1	169.0	169.5	169.0	169.5	169.0	169.0	169.0
Participant 2	165.0	165.3	165.0	165.1	165.4	165.3	165.1
Participant 3	170.0	170.1	169.8	170.0	170.2	169.7	170.2

Supplemental Table S1.2b. Interrater reliability of mass (kg) measurements recorded from 3 participants by each of the 7 researchers, ICC = 1.00

	Researcher 1	Researcher 2	Researcher 3	Researcher 4	Researcher 5	Researcher 6	Researcher 7
Participant 1	94.4	94.4	94.4	94.4	94.4	94.4	94.4
Participant 2	61	61	61	61	61	61	61
Participant 3	62.3	62.3	62.3	62.3	62.3	62.3	62.3

Supplemental Table S1.2c. Interrater reliability of arm girth (cm) measurements recorded from 3 participants by each of the 7 researchers, ICC = 0.993

	Researcher 1	Researcher 2	Researcher 3	Researcher 4	Researcher 5	Researcher 6	Researcher 7
Participant 1	36.9	36.3	35.5	36.2	36.4	36.5	36.4
Participant 2	28	27.7	28.5	27.5	28.3	28.2	28.4
Participant 3	25	26	25.3	25.4	24.9	25.2	24.8

Supplemental Table S1.2d. Interrater reliability of chest girth (cm) measurements recorded from 3 participants by each of the 7 researchers, ICC = 0.9896

	Researcher 1	Researcher 2	Researcher 3	Researcher 4	Researcher 5	Researcher 6	Researcher 7

Participant 1	99.2	97.9	98.3	98.1	99.1	98.4	97.9
Participant 2	89.5	88.5	87.8	87.6	88.9	87.6	88.0
Participant 3	84.3	82.8	82.5	83.8	86.2	85.1	84.9

Supplemental Table S1.2e. Interrater reliability of waist girth (cm) measurements recorded from 3 participants by each of the 7 researchers, ICC = 0.984

	Researcher 1	Researcher 2	Researcher 3	Researcher 4	Researcher 5	Researcher 6	Researcher 7
Participant 1	89.2	87.1	88.5	86.9	88.9	90.0	89.1
Participant 2	72.3	73.5	72.4	70.6	71.5	73.2	74.8
Participant 3	83.0	83.5	82.7	83.6	83.5	82.3	84.0

Supplemental Table S1.2f. Interrater reliability of hip girth (cm) measurements recorded from 3 participants by each of the 7 researchers, ICC = 0.9979

	Researcher 1	Researcher 2	Researcher 3	Researcher 4	Researcher 5	Researcher 6	Researcher 7
Participant 1	118.3	120.0	120.4	118.5	119.8	119.9	120.5
Participant 2	90.7	89.7	91.3	91.0	90.0	91.3	90.8
Participant 3	92.0	94.6	94.5	93.4	92.8	95.0	93.4

Supplemental Table S1.2g. Interrater reliability of thigh girth (cm) measurements recorded from 3 participants by each of the 7 researchers, ICC = 0.9945

	Researcher 1	Researcher 2	Researcher 3	Researcher 4	Researcher 5	Researcher 6	Researcher 7
Participant 1	73.1	74.5	73.3	73.0	73.4	72.5	73.5
Participant 2	53.2	53.5	55.2	54.8	54.2	54.8	54.4
Participant 3	60.4	59.6	59.4	60.0	60.2	59.4	59.0

Supplemental Table S1.2h. Interrater reliability of forced vital capacity (L) measurements recorded from 3 participants by each of the 7 researchers, ICC = 0.7927

	Researcher 1	Researcher 2	Researcher 3	Researcher 4	Researcher 5	Researcher 6	Researcher 7
Participant 1	4.13	4.13	4.13	4.14	4.07	4.14	4.06
Participant 2	3.70	3.65	3.81	3.81	3.81	3.79	3.79
Participant 3	3.84	4.18	4.05	4.01	3.96	4.25	4.19

2. Additional Results

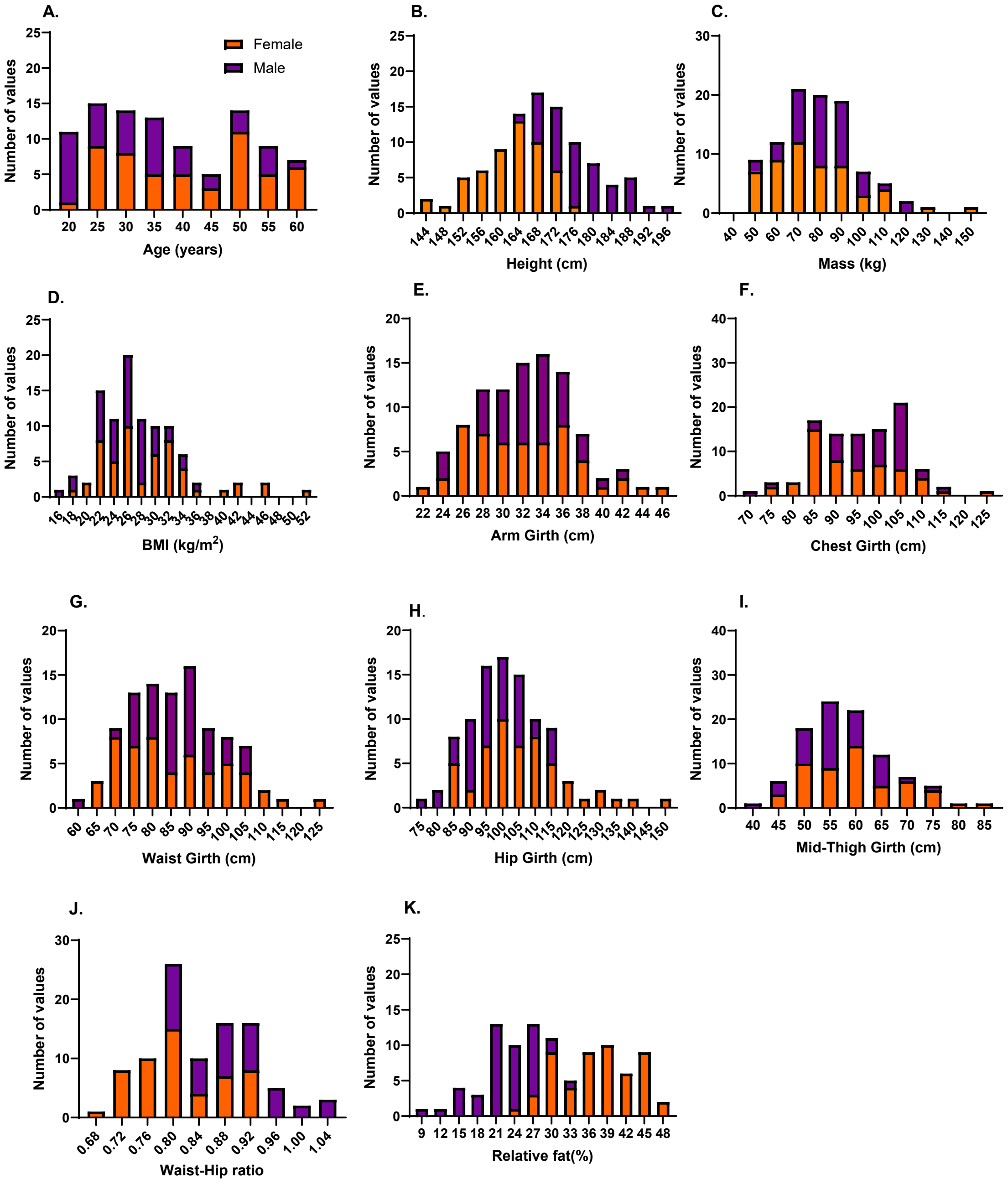

Figure S1.1 Frequency distributions of male (purple bars n=43) and female (orange bars n=53) participants for (A) age (years), (B) height (m), (C) mass (kg), (D) BMI (kg.m^{-2}), (E) arm (cm), (F) chest, (cm), (G) waist (cm), (H) hip (cm) and (I) thigh (cm) girths, (J) waist-to-hip ratio, and (K) relative fat equation (%)[1].

© Supplemental materials for Massey et al (2026) IJERPH

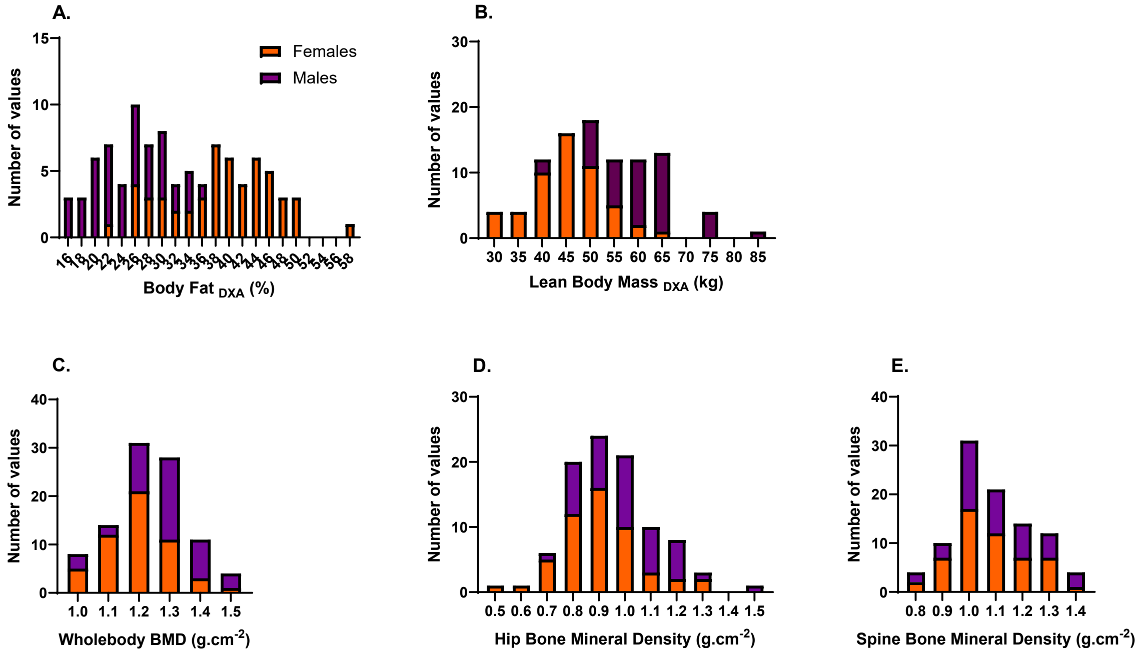

Figure S1.2 Frequency distributions of male (purple bars n=43) and female (orange bars n=53) participants for (A) DXA derived body fat (%), (B) lean body mass derived from DXA (kg), (C) whole body bone mineral density (g.cm^{-2}), (D) hip bone mineral density (g.cm^{-2}), (E) spine bone mineral density (g.cm^{-2}).

© Supplemental materials for Massey et al (2026) IJERPH

Float descriptions

Table S2.1. Frequency (percentages) of arm movements during the last 30 s of a 2 min float separated by float outcome.

Float Outcome	Frequency				Intensity			Work rest pattern				Size			Coordination		Technique				
	Fast	Mod-erate	Slow	No	High	Mod-erate	Low	Cont-inous	Bouts of activity	Inter-mediate	Occas-ional	Hands only	Hands and forearm	Whole arm	Sync	Alter-nating)	Scull	Circle	Push down	Flap /sweep	None
**Floats with no effort**				50 (52%)																	48 (50%)
**Floats with little effort**	3 (3%)	5 (5%)	8 (8%)	1 (1%)			16 (17%)	6 (6%)	4 (4%)	1 (1%)	5 (5%)	1 (1%)	1 (1%)	14 (15%)	16 (17%)		3 (3%)	6 (6%)	5 (5%)	6 (6%)	1 (1%)
**Floats with some effort**	5 (5%)	9 (9%)		1 (1%)	3 (3%)	11 (11%)		13 (14%)	1 (1%)					12 (13%)	14 (15%)		4 (4%)	5 (5%)	7 (7%)	3 (3%)	1 (1%)
**Floats with lots of effort**	7 (7%)	2 (2%)			7 (7%)	1 (1%)	1 (1%)	8 (8%)	1 (1%)					8 (8%)	9 (9%)		2 (2%)	4 (4%)	6 (6%)	1 (1%)	
**Cannot float**	3 (3%)	4 (4%)			1 (1%)	2 (2%)	4 (4%)	6 (6%)			1 (1%)			6 (6%)	6 (6%)		3 (3%)	3 (3%)		2 (2%)	

The percentages will not add up to 100% as several participants adopted more than one arm action during the analysis period

© Supplemental materials for Massey et al (2026) IJERPH

Table S2.2. Frequency (percentages) of leg action during the last 30 s of a 2 min float separated by float outcome.

**Float Outcome**	**Frequency**				**Intensity**			**Work rest pattern**				**Size**			**Coordination**		**Technique**			
	**Fast**	**Mod-erate**	**Slow**	**No**	**High**	**Mod-erate**	**Low**	**Cont-inous**	**Bouts of activity**	**Inter-mediate**	**Occas-ional**	**Feet**	**Feet and lower leg**	**Whole leg**	**Sync**	**Alter-nating**	**Cycle**	**Flutter /flick**	**Breaststroke /egg beater**	**None**
**Floats with no effort**				48 (50%)																48 (50%)
**Floats with little effort**	1 (1%)	3 (3%)	4 (4%)	9 (9%)			8 (8%)	6 (6%)			2 (1%)		5 (5%)	3 (3%)		8 (8%)	8 (8%)	2 (2%)		9 (9%)
**Floats with some effort**	2 (2%)	7 (7%)		4 (4%)	1 (1%)	6 (6%)	3 (3%)	9 (9%)	1 (1%)					10 (10%)	2 (2%)	8 (8%)	7 (7%)	2 (2%)	2 (2%)	4 (4%)
**Floats with lots of effort**	3 (3%)	1 (1%)		5 (5%)	2 (2%)	2 (2%)		4 (4%)						4 (4%)		4 (4%)	1 (1%)	2 (2%)		5 (5%)
**Cannot float**	1 (1%)			6 (6%)	1 (1%)			1 (1%)						1 (1%)		1 (1%)	1 (1%)			6 (6%)

Mod = moderate, Cont = continuous,

The percentages will not add up to 100% as several participants adopted more than one leg action during the analysis period

© Supplemental materials for Massey et al (2026) IJERPH

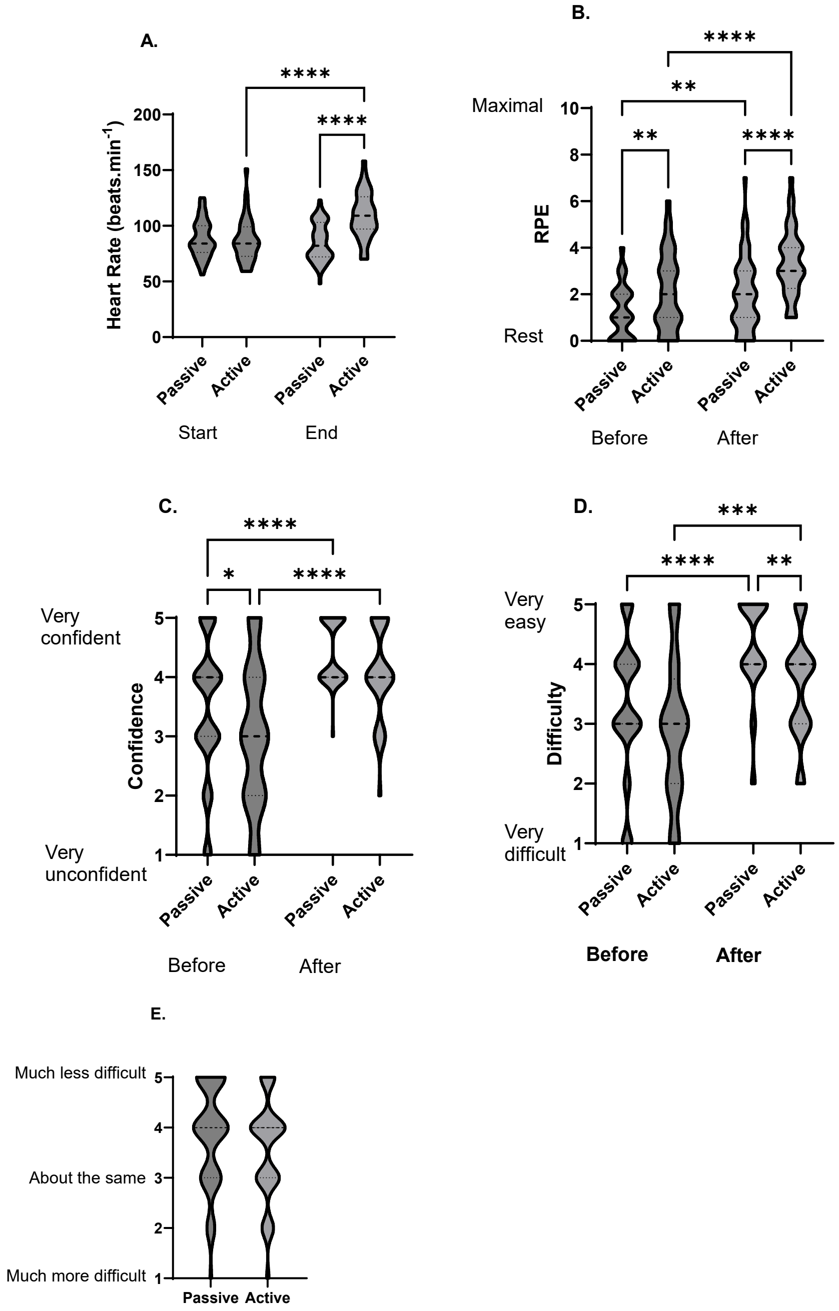

Figure S1.3. Responses to floating in participants who floated actively or passively. A. Heart rate at the start of the float and end of the float, B. rate of perceived exertion, C. confidence, D. difficulty before and immediately after the float, and E. the perception of difficulty in response to the question: How did floating compare to how difficult you thought it would be? The width of the violin plots indicates the frequency of votes. Bars indicate significant difference, * P <0.05, **P <0.01, *** P <0.001, ****P <0.0001.

© Supplemental materials for Massey et al (2026) IJERPH

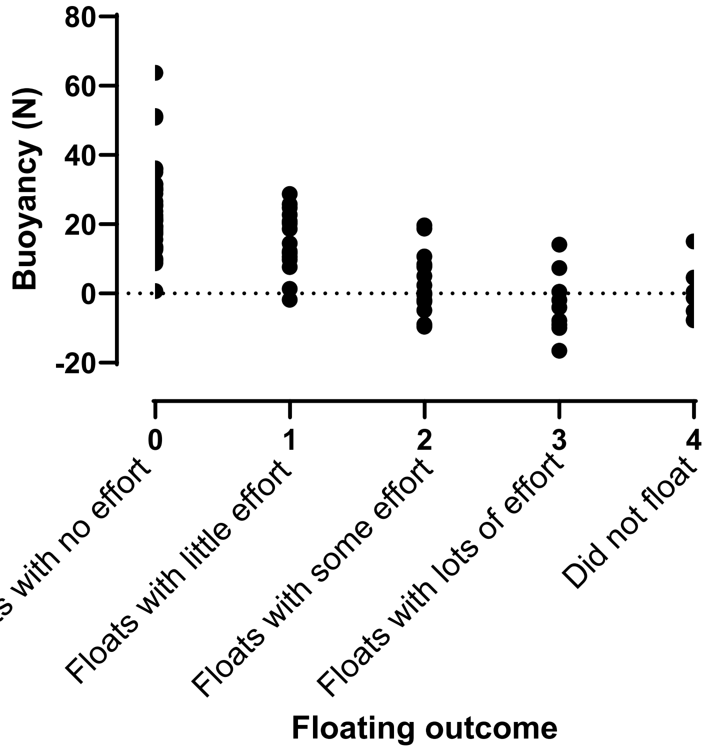

Figure S1.4 Relationship between float outcome and buoyancy.

© Supplemental materials for Massey et al (2026) IJERPH

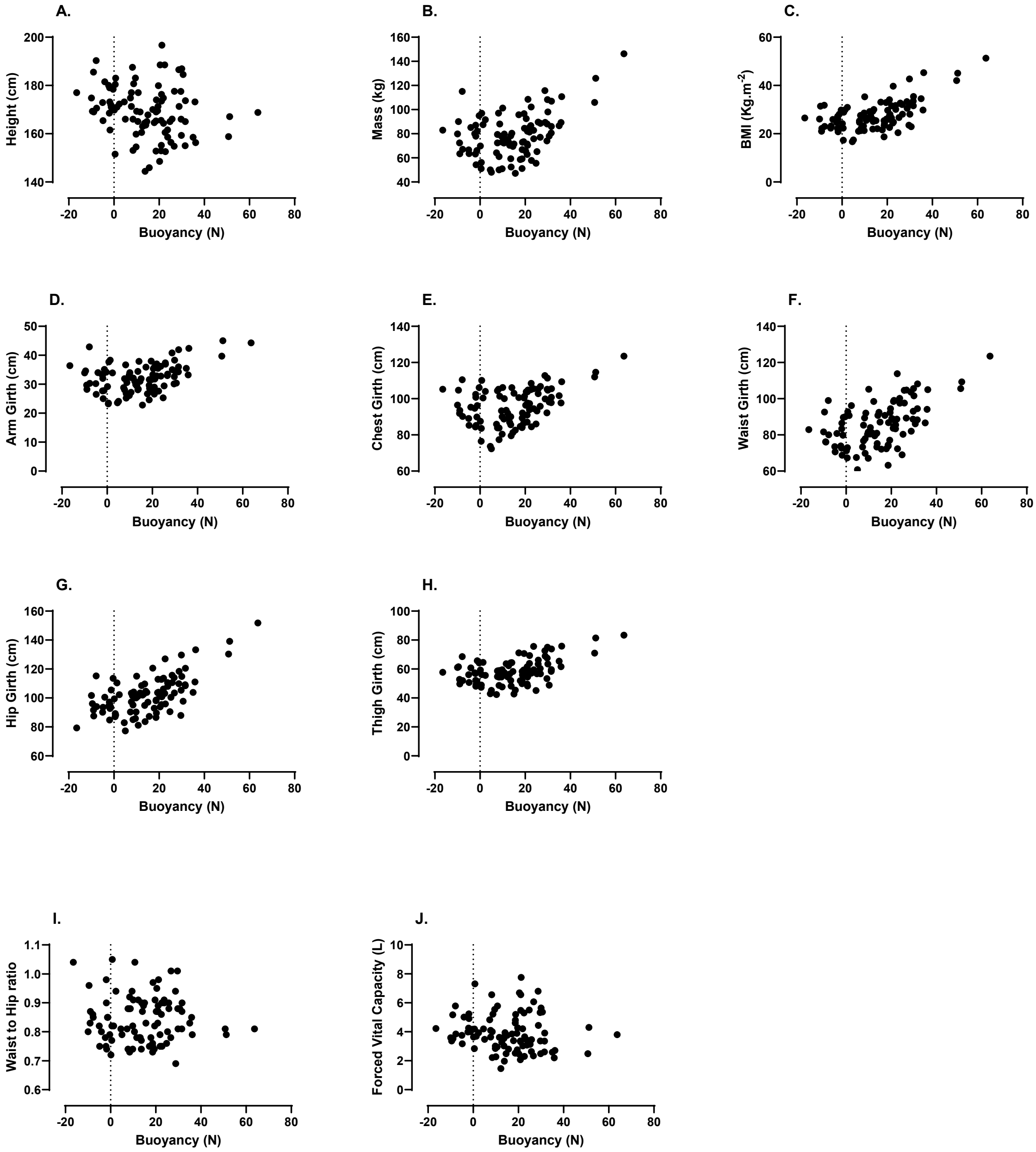

Figure S1.5. Anthropometric and physiologic measurements correlated against buoyancy. A, Height (cm); B, mass (kg); C, Body Mass Index (kg.m^{-2}); D, arm circumference (cm); E, chest circumference (cm); F, waist circumference (cm); G, hip circumference (cm); H, thigh circumference (cm); I, waist-to-hip ratio; J, forced vital capacity (L).

© Supplemental materials for Massey et al (2026) IJERPH

Body Fat

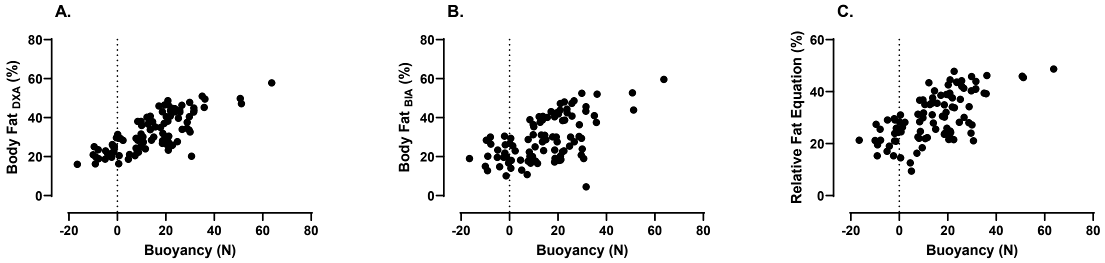

Lean Mass

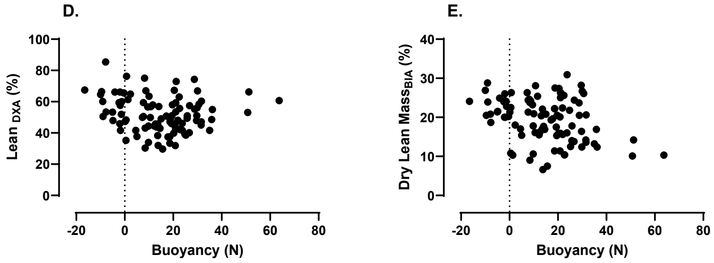

Android & Gynoid Fat

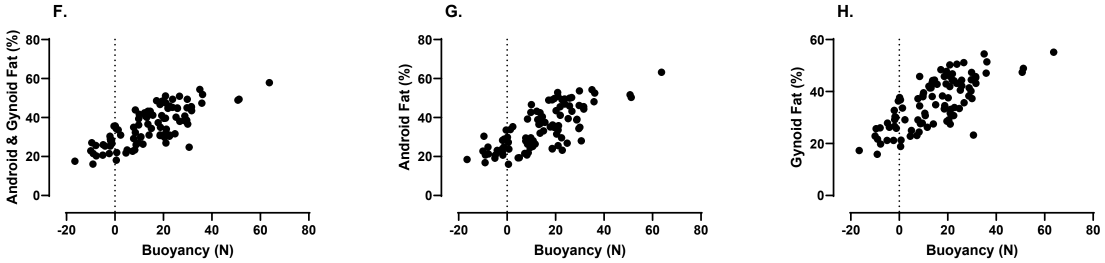

Bone Mineral Density

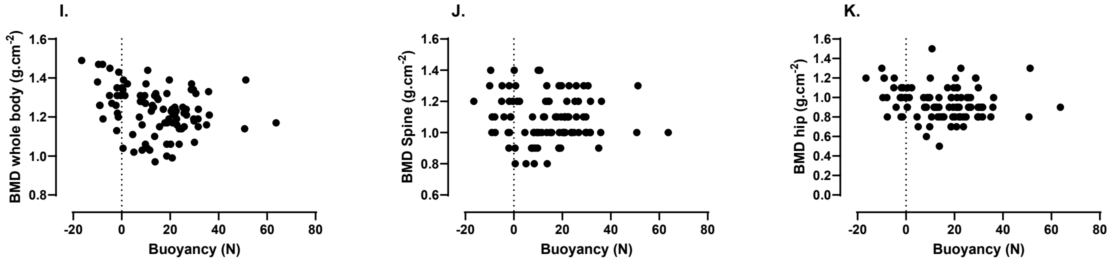

Figure S1.6. Body composition measurements correlated against Buoyancy. A, Body fat (%) measured from DXA scans; B, body fat (%) measured from Bio-electrical impedance; C, body fat (%) calculated from relative fat equations[1]; D, lean (%) wet measured by DXA; E, dry lean (%) dry measured by Bio-electrical impedance; F, Android and Gynoid fat (%); G, Android fat (%); H, Gynoid fat (%); I, bone mineral density of the whole body (g.cm^{-2}); J, bone mineral density of the spine (g.cm^{-2}); K, bone mineral density of the hip (g.cm^{-2}).

© Supplemental materials for Massey et al (2026) IJERPH

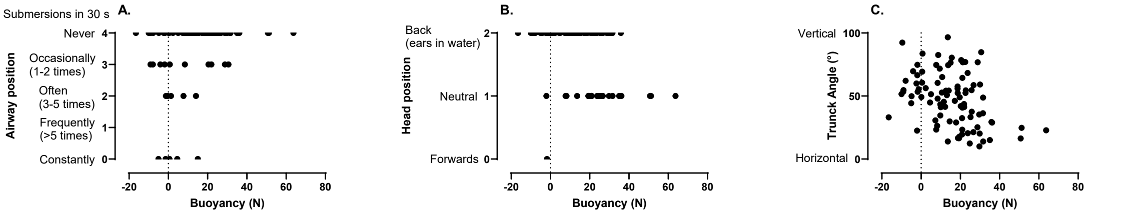

Arms

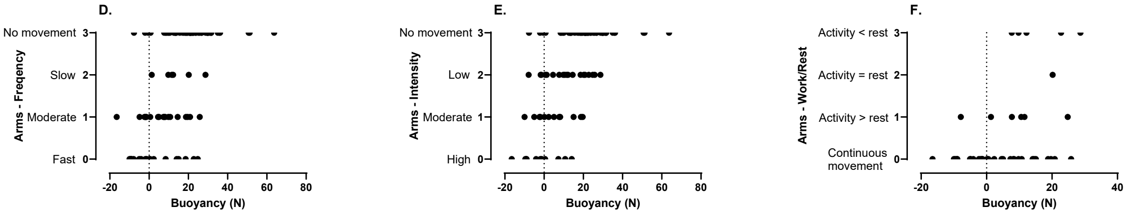

Legs

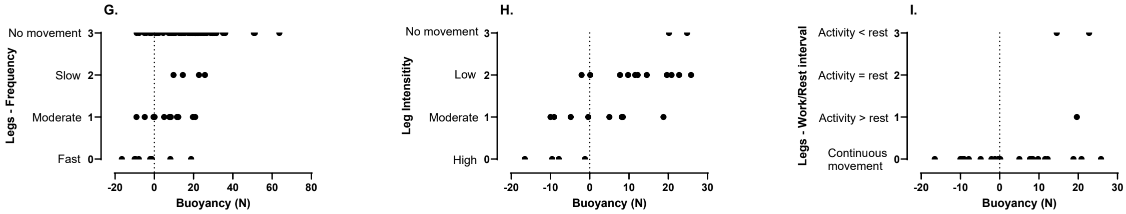

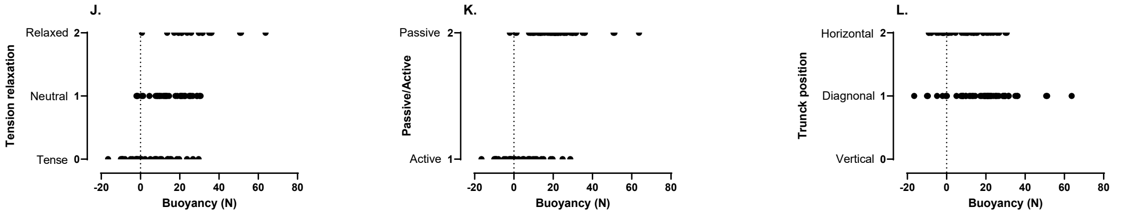

Figure S1.7 Floatation descriptors correlated against buoyancy. A, Airway position; B, head position; C, trunk angle (measured,°); D, arm movement frequency; E, arm movement intensity; F, arm movement work rest intervals; G, leg movement frequency; H, leg movement intensity; I, leg movement work rest intervals; J, tension relaxation; K, passive or active floatation; L, trunk position.

© Supplemental materials for Massey et al (2026) IJERPH

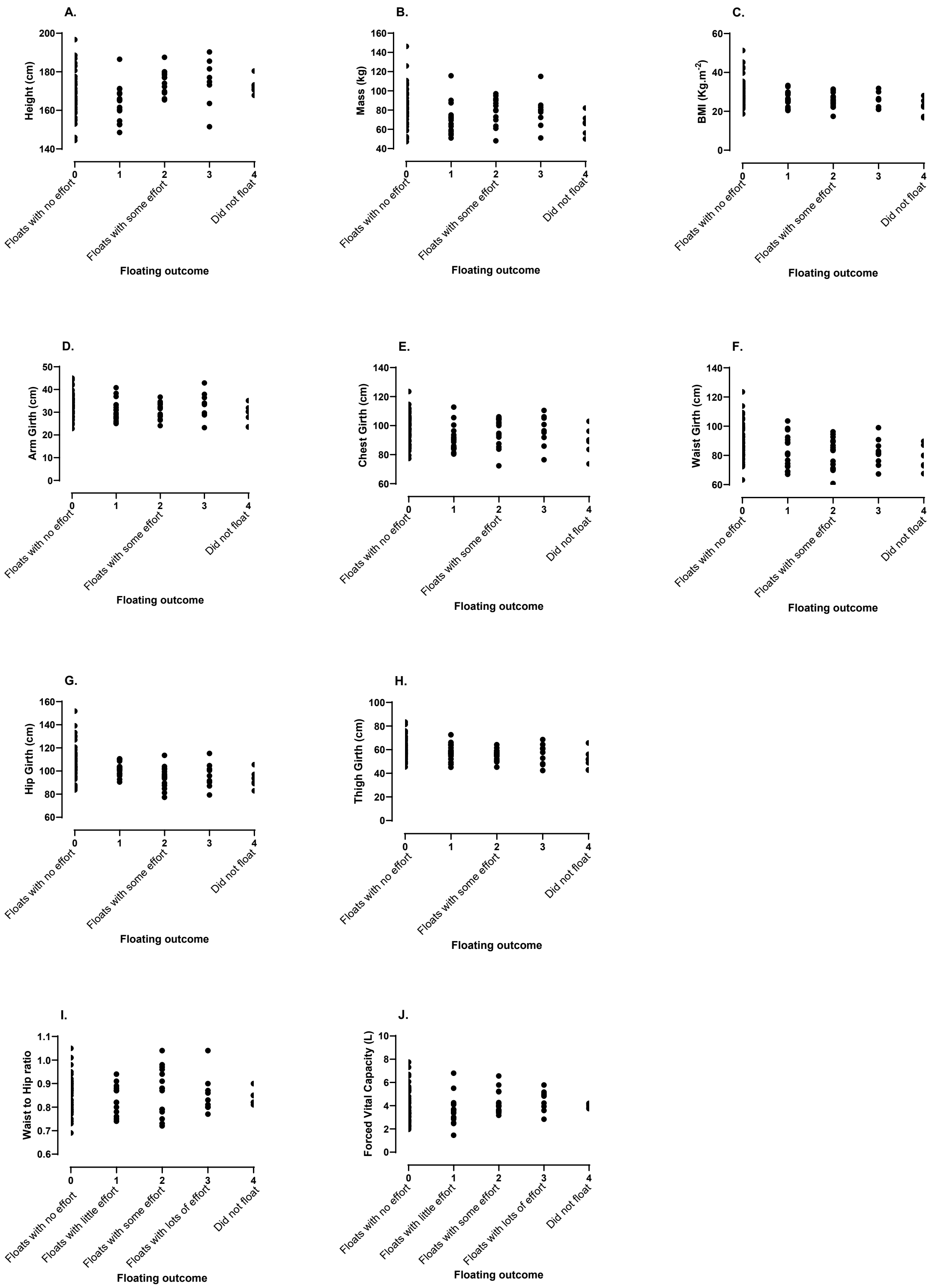

Figure S1.8 Anthropometric and physiologic measurements correlated against float outcome. A, Height (cm); B, mass (kg); C, Body Mass Index (kg.m^{-2}); D, arm circumference (cm); E, chest circumference (cm); F, waist circumference (cm); G, hip circumference (cm); H, thigh circumference (cm); I, waist-to-hip ratio; J, forced vital capacity (L).

© Supplemental materials for Massey et al (2026) IJERPH

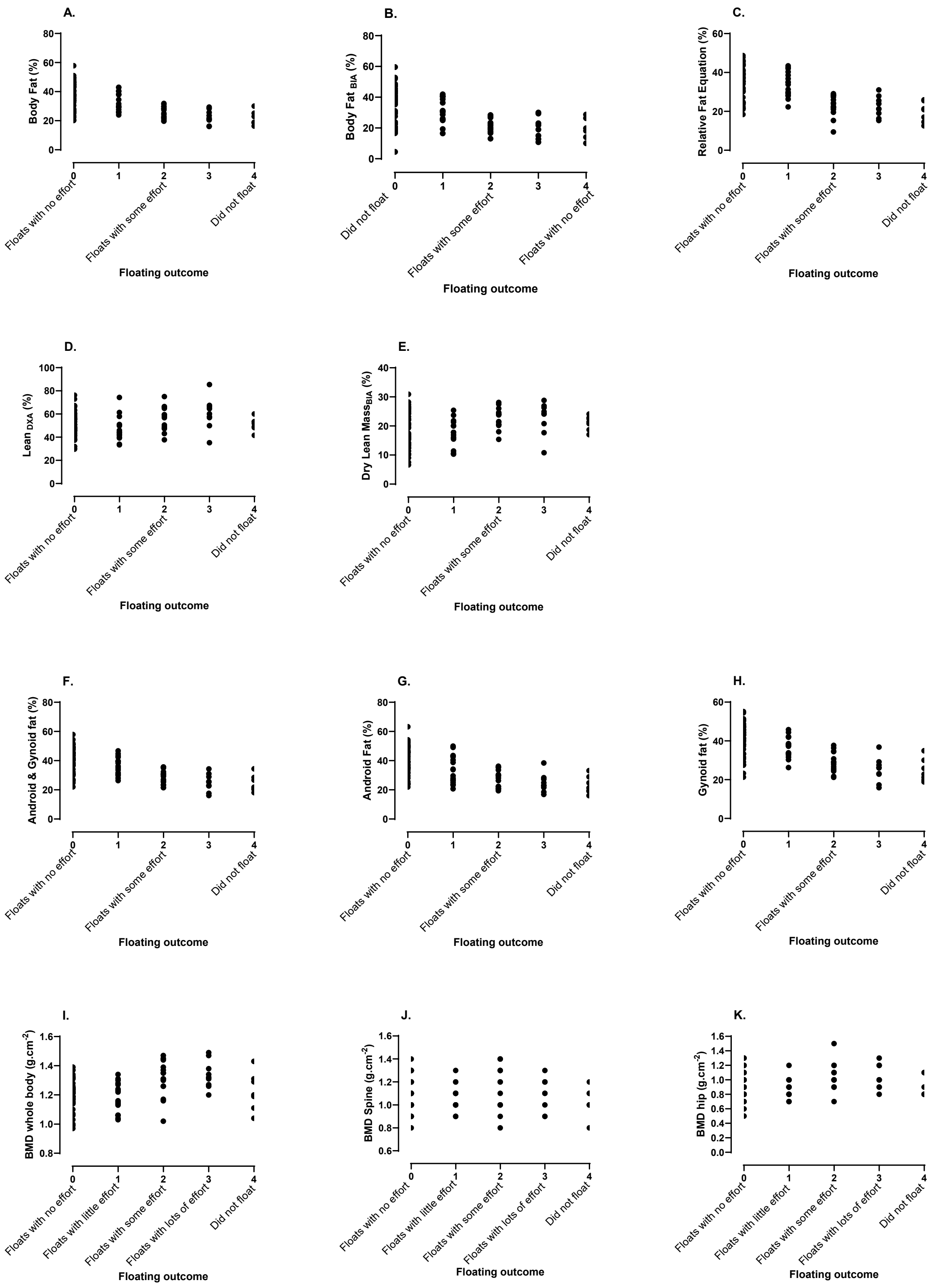

Figure S1.9. Body composition measures correlated against Float outcome. A, Body fat (%) measured from DXA scans; B, body fat (%) measured from Bio-electrical impedance; C, body fat (%) calculated from relative fat equations[1]; D, lean (%) wet measured by DXA; E, dry lean (%) dry measured by Bio-electrical impedance; F, Android and Gynoid fat (%); G, Android fat (%); H, Gynoid fat (%); I, bone mineral density of the whole body ($g.cm^{-2}$); J, bone mineral density of the spine ($g.cm^{-2}$); K, bone mineral density of the hip ($g.cm^{-2}$).

© Supplemental materials for Massey et al (2026) IJERPH

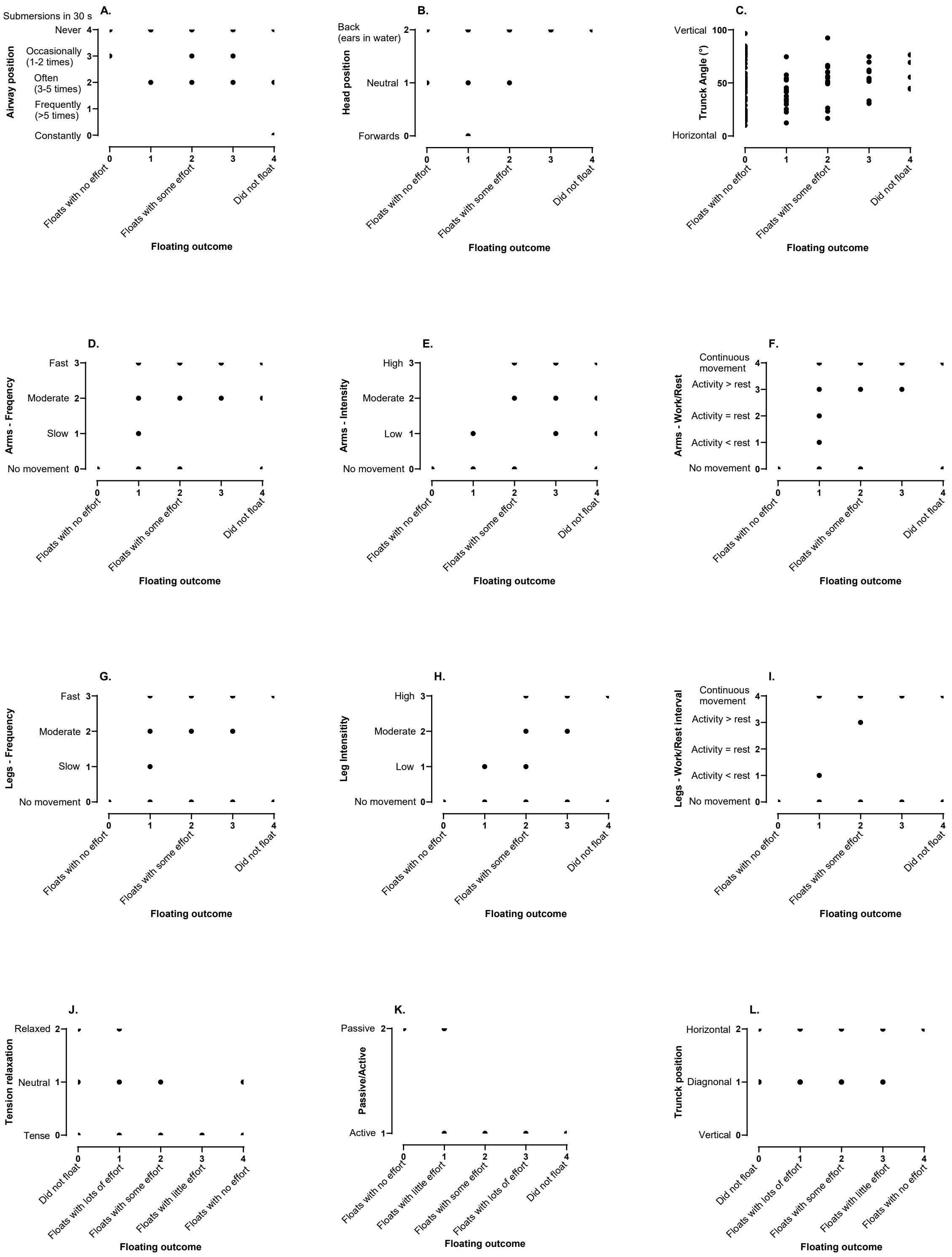

Figure S1.10 Floatation descriptors correlated against float outcome. A, Airway position; B, head position; C, trunk angle (measured,°); D, arm movement frequency; E, arm movement intensity; F, arm movement work rest intervals; G, leg movement frequency; H, leg movement intensity; I, leg movement work rest intervals; J, tension relaxation; K, passive or active floatation; L, trunk position.

© Supplemental materials for Massey et al (2026) IJERPH

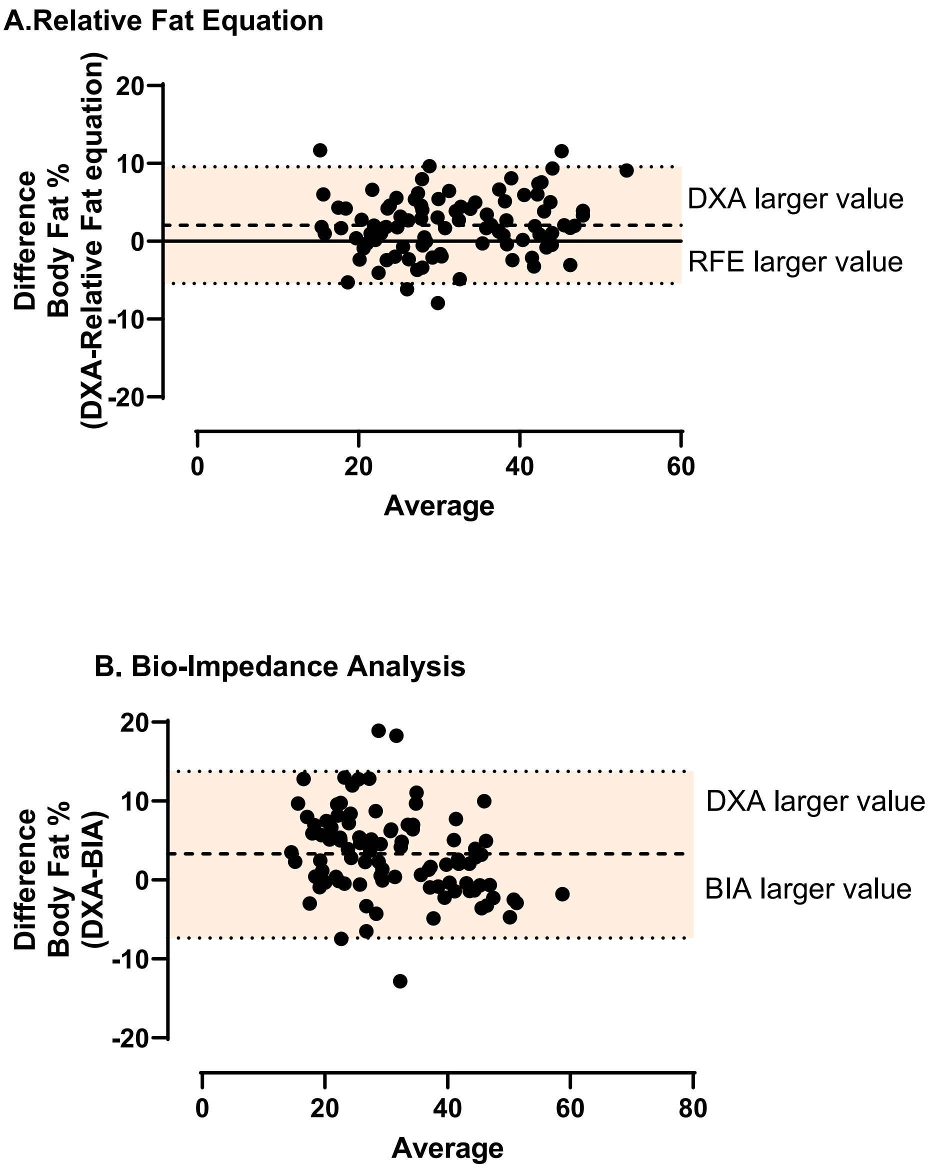

Supplemental Figure S1.11. Limits of agreement for body fat measurement methods. (A) DXA and BIA; (B) DXA and relative fat equation[1].

© Supplemental materials for Massey et al (2026) IJERPH

3. Qualitative analysis

Participants' comments about the float sessions

Prior to joining the study, participants were supported by representatives of the Black swimming association, who have extensive experience of supporting people from African, Caribbean and Asian communities who were non- or minimally aquatically active to learn to swim through their accredited Blue Hour/Together We Can programme. This support ranged from practical support over the phone, advising people what to bring with them, what to expect or helping them find the location and navigate the facilities. In the BSA's experience, these are all barriers people perceive, and their support and knowledge to aid participants in their journey into the study was invaluable. This expertise, founded on the BSA programme staff and work, was also apparent from the approach taken in the pool.

The instructions participants found best helped them float were: to relax (n = 51); put their head back (n = 50); and maintain shallow, consistent breathing /not breathing in or out too deeply or quickly (n = 49). Instructions on arm activity were found helpful by 34 people, while 23 found the guidance on leg movement helpful. Fourteen participants found it useful to try different floating positions/shapes, including floating in a prone position first; in addition, 11 participants suggested that practice was helpful, or they recognised that they were overthinking the activity, or they needed to remain calm. Seven participants explicitly mentioned that it was helpful to move, whether it was their location in the water or moving their arms or legs

Narrative accounts of instructors' processes to support people to float

The instructors also provided insight into the processes they used to support participants to float. An overview of their processes is provided in Figure 4.1, and a narrative account is provided below.

Whilst most of the comments from the participants described specific instructions that they found helpful to float, the instructors first tried to instil trust and calm: "*We build up a level of trust with them [participants], so my aim is to make them feel relaxed, to make them calm, to make them feel safe*". Several instructors indicated that through this relaxation, participants could better understand the effect the water had on their body and how they may be able to use their buoyancy to support their float "*You want them to connect with the water, because the only way to relax is to feel the water, how the water moves and the water is going to move your body as well when you relax. When you are tense, you don't get that connection of feeling held*".

© Supplemental materials for Massey et al (2026) IJERPH

After that, instructors focussed on the need to maintain regular breathing and feelings of confidence if their face becomes submerged " *I get them to start with getting their face in the water just so I can see that they can do that and blow bubbles so if their face goes under it's not going to be an immediate panic and they know what to do*". Then experiment with how their breathing changes their buoyancy "*showing the participants how important their breath is, is so important, the very first thing I actually do with them is get them to take a full inhalation, hold it and get a sense of their own buoyancy in the water and even also experimenting with expelling a bit of air too so they can see how their body sort of sinks and then comes up but not necessarily in the float position initially*". Several instructors discussed floating in a prone position first, "*I always like for someone to start on their front so they get that feeling of how it is feels to float because everyone seems to be more comfortable with holding their breath lying on their front and feeling that weightlessness in water that you can actually do it and then turn them over.*" Subsequent actions that help participants to stay afloat included the arm and leg actions: "*I have to teach them how to scull properly…how they have to catch the water*"; "*you get them to explore which position is comfortable for them and how much effort they need to put in with their arms and legs to stay afloat*".

Barriers participants felt to floating were also discussed: "*it's the fear of what might happen if I go under and I don't come back up. It's sort of a mental block for people more than a lack of physical capability*" "*when you go straight into a float on your back they've made lots of preconceived ideas that the float should be quite a horizontal and they try to go straight into that unsuccessfully, splashing water in their face, nose*". The instructors also explained how they gave information "*in small chunks, which was gently reinforced, with lots of praise when the participant responded*" "*must remember that giving lots of information in one go is not ideal, take it incrementally, when one action has been achieved consistently build on that with new information".* All instructors suggested that trial and error was appropriate given the controlled nature of the environment *"don't be afraid to try different techniques until you fall on a shape that feels good for your swimmer, then build from there" and the opportunity to experiment with what works best for a person "if they don't float completely on the surface in a star shape, find what does work for them"*.

References

1. Woolcott OO, Bergman RN. Relative fat mass (RFM) as a new estimator of whole-body fat percentage – A cross-sectional study in American adult individuals. *Sci Rep*. 2018;8(1):10980. doi:10.1038/s41598-018-29362-1

© Supplemental materials for Massey et al (2026) IJERPH
